# Homologous Recombination Repair Mechanisms in Serous Endometrial Cancer

**DOI:** 10.3390/cancers13020254

**Published:** 2021-01-12

**Authors:** Jenny-Maria Jönsson, Maria Bååth, Ida Björnheden, Irem Durmaz Sahin, Anna Måsbäck, Ingrid Hedenfalk

**Affiliations:** 1 Division of Oncology, Department of Clinical Sciences, Lund University, 223 81 Lund, Sweden; maria.baath@med.lu.se (M.B.); irsahin@ku.edu.tr (I.D.S.); ingrid.hedenfalk@med.lu.se (I.H.); 2 Department of Oncology, Skåne University Hospital, 221 85 Lund, Sweden; 3 Department of Surgical Pathology, Skåne University Hospital, 221 85 Lund, Sweden; ida.bjornheden@skane.se (I.B.); anna.masback@skane.se (A.M.); 4 Department of Molecular Biology and Genetics, Koc University School of Medicine, 344 50 Istanbul, Turkey

**Keywords:** serous endometrial cancer, homologous recombination repair deficiency, DNA repair, copy-number variation, PARP inhibition

## Abstract

**Simple Summary:**

Serous endometrial cancer is an unusual and aggressive endometrial cancer subtype, conferring the highest mortality of all endometrial cancers. In many ways, it resembles the more common tumor entity high-grade serous ovarian cancer. Thus, there is an urgent need for better treatment options for serous endometrial cancer patients. It is crucial for all dividing cells that the DNA repair is functioning correctly. Our aim was to investigate deficiencies in DNA repair in serous endometrial cancer, in particular the presence of homologous recombination repair deficiency. This kind of DNA repair defect may indicate that a specific targeted therapy, so-called PARP inhibitors, which are already in use for the treatment of ovarian cancer, may be useful also in serous endometrial cancer. This study contributes to the largely unexplored field of DNA repair deficiencies in serous endometrial cancer, and may hence contribute to future improved prognosis for these patients.

**Abstract:**

Serous endometrial cancer (SEC) resembles high-grade serous ovarian cancer (HGSOC) genetically and clinically, with recurrent copy number alterations, *TP53* mutations and a poor prognosis. Thus, SEC patients may benefit from targeted treatments used in HGSOC, e.g., PARP inhibitors. However, the preclinical and clinical knowledge about SEC is scarce, and the exact role of defective DNA repair in this tumor subgroup is largely unknown. We aimed to outline the prevalence of homologous recombination repair deficiency (HRD), copy-number alterations, and somatic mutations in SEC. OncoScan SNP arrays were applied to 19 tumors in a consecutive SEC series to calculate HRD scores and explore global copy-number profiles and genomic aberrations. Copy-number signatures were established and targeted sequencing of 27 HRD-associated genes was performed. All factors were examined in relation to HRD scores to investigate potential drivers of the HRD phenotype. Ten of the 19 SEC tumors (53%) had an HRD score > 42, considered to reflect an HRD phenotype. Higher HRD score was associated with loss of heterozygosity in key HRD genes, and copy-number signatures associated with non-*BRCA1/2* dependent HRD in HGSOC. A high number of SECs display an HRD phenotype. It remains to be elucidated whether this also confers PARP inhibitor sensitivity.

## 1. Introduction

Serous endometrial cancer (SEC) is a both rare and aggressive subtype of endometrial cancer (EC). Constituting 10–15% of all ECs, but with a relative five-year survival of only 50%, SEC accounts for approximately 40% of all EC deaths [1,2]. Like many other malignancies, EC is a heterogeneous disease and can be divided into not only histological but also molecular subtypes. Based on somatic copy-number alterations and exome sequencing, the EC molecular subtypes are stratified into the *POLE* ultra-mutated, microsatellite instability/hypermutated, copy-number high/serous-like and copy-number low subtypes, respectively [3]. Later refinements have resulted in immunohistochemistry (IHC) surrogates, with, e.g., the copy-number high/serous-like subtype corresponding to *TP53* aberrant tumors [4,5,6]. SEC almost exclusively falls into the copy-number high/serous-like subtype, with a high frequency of copy-number alterations and *TP53* mutations and few *PTEN* mutations, corresponding with a worse survival. Thus, SEC resembles its namesake high-grade serous ovarian cancer (HGSOC), both clinically and molecularly, and the molecular subtypes have the potential to influence adjuvant treatment choices [3,4,5,7].

The poly (ADP-ribose) polymerase (PARP) enzymes are involved in repair of single-strand DNA breaks, and inhibition of PARP leads to impaired single-strand repair and consequently to the formation of double-strand breaks. Defects in homologous recombination repair (HR) genes used to repair double-strand breaks, e.g., mutations in *BRCA1*, *BRCA2*, and *RAD51,* lead to HR deficiency (HRD), and confer sensitivity to PARP inhibition. The genomic scars caused by HRD can be observed by loss of heterozygosity (LOH), telomeric allelic imbalance (TAI), and large-scale state transitions (LST), which can be quantified separately or together as a measurement of the HRD phenotype [8,9,10,11]. There is convincing evidence that approximately 15% of HGSOCs harbor germline *BRCA1/2* mutations and possibly as many as 50% display an HRD phenotype [12]. The associations between HRD, PARP inhibition, *TP53* mutations, and platinum sensitivity in HGSOC are well established [13]. However, even other types of DNA repair than HRD, including Non-Homologous End Joining (NHEJ), Base Excision Repair (BER), Nucleotide Excision Repair (NER), and Mismatch Repair (MMR), may be of importance since a subset of HR proficient HGSOCs also respond to PARP inhibitors [14]. The knowledge in this area in SEC, however, is limited. The potential connection between *BRCA1/2* mutations and development of EC is debated, and data are somewhat contradictory [7,15,16]. A large study on HRD frequency in solid tumors revealed HRD in >30% of ECs, and in limited cohorts HRD has been found in 30–50% of SECs, primarily due to *RAD51* mutations [17,18,19]. Thus, defective DNA repair, including HRD, is most certainly important in SEC, but its exact role remains to be elucidated. So-called mutational signatures, whereby combinations of somatic mutation types are categorized, may provide information about oncogenic processes involved in disease development and progression. An HRD-related mutational signature has been established, but, surprisingly, has only been reported to occur in approximately 15% of SECs. This may imply that only a minority of SECs would actually benefit from PARP inhibitor treatment [20,21]. Recently, a computational method was developed, using shallow whole-genome sequencing data from HGSOC cases, with the purpose of condensing copy-number data into levels of exposure to seven different copy-number signatures [22]. This method may be applied also to other cancer forms, including SEC, to distinguish tumors with different genomic profiles and potentially also different treatment responses.

The need for improved treatment options in SEC is urgent, and enhanced knowledge about HRD as well as better biomarkers for HRD in SEC are promising contributions. Therefore, our aim was to outline the prevalence of an HRD phenotype in a consecutive SEC cohort and to analyze the genomic landscape and somatic mutations in HRD-associated genes, with the purpose to try to reveal a subgroup of SECs that may respond to PARP inhibitors or other targeted treatments.

## 2. Materials and Methods

### 2.1. Patient and Tumor Characteristics

A consecutive series of 31 SECs was collected from our local tissue biobank (Region Skåne’s biobank for gynecological tumors, Lund, Sweden), containing both fresh-frozen and formalin-fixed paraffin-embedded (FFPE) tumor tissue, and whole blood, from March 2015 to July 2016. Histologic subtype was determined according to WHO 2014 [23] and all tumors were staged according to the International Federation of Gynecology and Obstetrics (FIGO) 2009 criteria [24]. All cases underwent expert pathology review, and two cases were removed due to revised diagnosis. Of the remaining 29 cases, insufficient amount of tissue for DNA extraction (*n* = 4), or a low percentage of tumor cells in the specimen (*n* = 6) was present, leaving 19 cases for further analyses. Tumor samples were collected when performing diagnostic endometrial biopsies or at primary surgery, and the patients had not received prior chemotherapy. Twelve out of 19 (63%) tumors were diagnosed in stage I. No patients had known mutations in *BRCA1*, *BRCA2*, or in the MMR genes. The median follow-up time was 47 months (range 2–60 months). Brief clinical data are outlined in Table 1, and detailed data are available in Supplementary Table S1. The study was conducted in accordance with the declaration of Helsinki, and ethical approval for the study was granted from the Lund University ethics committee, Sweden (EPN 2016/508). All patients provided written informed consent to participate in the study.

### 2.2. Global Copy-Number Analyses

DNA was extracted from FFPE tissue. Extractions were performed using the Qiagen AllPrep kit for FFPE tissue (Qiagen^®^, Venlo, The Netherlands). 80 ng DNA was used as input for single nucleotide polymorphism (SNP) array analysis at Eurofins Genomics Europe Genotyping A/S (Galten, Denmark, The Netherlands) using the OncoScan^®^ FFPE Assay Kit (Affymetrix^®^, Santa Clara, CA, USA). After preprocessing using the manufacturer’s standard protocol, segmentation was performed using ASCAT (package version 2.5.2, in R version 3.6.3). With this package, we also derived copy-number profiles of tumor cells and estimates of normal cell contamination and ploidy. The fraction of the genome altered (FGA) was calculated using fraction of probe positions with a total copy-number differing from ploidy by >0.6.

### 2.3. Analyses of HRD and DNA Double-Strand Break Repair Genes

An HRD score was calculated from output of ASCAT using implementations in R as described by Telli et al. [8]. Estimates of the global levels of LOH, LST, and TAI were calculated separately, and the unweighted sum of these was defined as the HRD score. A score of >42 was used as the cut-off for HRD, as defined in breast cancer and previously applied also to HGSOC [8]. A previously described panel of 102 “core” and “related” HRD genes was used to investigate whether specific HRD genes, or a certain number of these genes, correlated with the HRD scores [25]. A smaller panel of NHEJ core genes was also investigated in order to get a broader picture of each tumor’s DNA double-strand break repair system [26,27]. The genes coding for the following eight factors were included: tumor protein p53 binding protein 1 (*TP53BP1*), Ku70 (*XRCC6*), Ku80 (*XRCC5*), DNA-dependent protein kinase catalytic subunit (DNA-PKcs, *PRKDC*), DNA Ligase IV (*LIG4*), X-ray repair cross-complementing protein 4 (*XRCC4*), XRCC4-like factor (*XLF*) and replication timing regulatory factor 1 (*RIF1*).

### 2.4. Gene Amplifications and Deletions

The segmented copy-number data generated through ASCAT were used to define gain, loss, amplification, homozygous deletion and LOH. Genomic Identification of Significant Targets in Cancer (GISTIC) version 2.0.23 was used to identify amplification peaks and to find driver genes. Detailed information is available in Appendix A.

### 2.5. Targeted Sequencing

A panel of 27 genes associated with hereditary breast and ovarian cancer and Lynch-associated cancers was sequenced at CaPreDx^®^, Lund, Sweden to determine the potential role of somatic mutations in these genes in relation to HRD status in our cohort. Detailed data on targeted sequencing is available in Appendix A. Data were filtered to exclude germline variants, common variants and mutations previously reported as benign or likely benign by ClinVar (30 May 2020) [28]. Only variants reported as known pathogenic or likely pathogenic were included in the analysis. All discovered variants are presented in Supplementary Table S2.

### 2.6. Copy-Number Signatures

Next, HGSOC-based copy-number signatures derived from whole-genome sequencing were analyzed. Exposure to each specific signature displays the fraction (out of 1) of the copy-number profile explained by that signature. We implemented the calculations in our cohort using copy-number information from segmented SNP-array data obtained from ASCAT. Calculations were performed using R-code from the depository of Macintyre et al. [22].

### 2.7. Immunohistochemistry (IHC)

Growth pattern and morphologic characteristics were evaluated using hematoxylin & eosin (H&E)-stained sections. H&E-stained 4 μm tumor sections were also used to detect viable tumor areas before DNA extraction. IHC staining was conducted to improve diagnostic reliability, with a special focus on p53, PTEN, and the mismatch repair proteins MLH1, MSH2, MSH6, and PMS2. A detailed description of the antibodies, staining and evaluation criteria is available in Supplementary Table S3. All IHC stainings were evaluated by IB and AM using whole tissue sections. Potential associations between the IHC markers were analyzed, and IHC stainings of p53 and PTEN were compared with sequencing data for the corresponding genes.

### 2.8. Statistics

Analyses were primarily focused on visual examination of the data. Statistical testing was performed only when differences between groups or correlations between variables appeared plausible upon visual inspection of plots, alternatively were warranted due to previously reported significance of specific genes. Pearson correlation was used for analysis of the pairwise association between two continuous variables and is presented with a correlation coefficient (r) and 95% confidence intervals (CIs). Welsh’s t-test was used to analyze the differences in mean of a continuous variable between two groups. All calculations and analyses were conducted using R statistical environment version 3.6.3 [29], except the statistical analyses used for scoring of IHC markers, which were performed using the *χ*^2^ test in SPSS (version 24.0). Overall survival was evaluated in relation to HRD using the Kaplan-Meier estimator.

## 3. Results

### 3.1. Global Copy-Number Analyses

**Figure 1:**
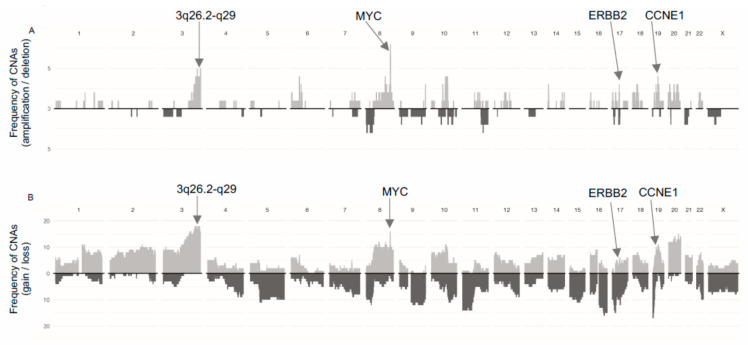
Frequency of Copy-Number Alterations (CNAs) sorted by (**A**) amplifications/deletions, and (**B**) gains/losses in the 19 tumors. Positions of frequently occurring driver genes are indicated with arrows [3,30]. Bars directed upwards represent amplifications/gains and bars directed downwards represent deletions/losses.

A summary of the frequency of gains/losses and amplifications/deletions is presented in Figure 1. Ten of the 19 tumors were aneuploid (>2.7n) with mean aberrant cell fractions of 52% (range 25–79%). The mean FGA was 52% (range 8–96%) and was not related to HRD score (r = 0.2, *p* = 0.36).

### 3.2. HRD and HRD-Associated Genes

**Figure 2:**
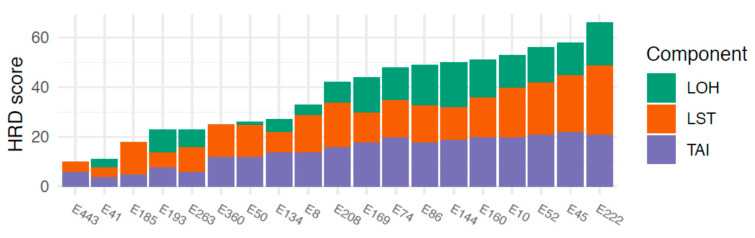
HRD scores and the respective underlying components. LOH = loss of heterozygosity, LST = large-scale state transitions, TAI = telomeric allelic imbalance. Cases are sorted by increasing HRD score along the *x*-axis.

HRD scores ranged between 10 and 66. An HRD score of >42, defined as the threshold for HRD, was observed in 10/19 (53%) tumors. The three components comprising the combined HRD score (LOH, AI, LST) all contributed to a fairly equal extent to the final HRD scores, showing that the HRD score was not driven by any single component (Figure 2).

In order to investigate if genomic alterations in any specific genes contributed to the wide range of HRD scores, 102 previously defined HRD-associated genes (40 “core” and 62 “related” genes) [25] were examined for genomic loss, LOH, and homozygous deletion in relation to HRD scores. From this gene panel, we observed that the number of genes with LOH was positively correlated with HRD score (r = 0.71 (0.37; 0.88), *p* < 0.001, Supplementary Figure S1A). The number of genes with genomic loss did not appear to correlate with HRD score (r = 0.39, (−0.077; 0.72), *p* = 0.098, Supplementary Figure S1B). Among these genes, *BRCA1* was one of the genes most frequently displaying aberrations, with an LOH frequency of 13/19 tumors, and genomic loss in 12/19 tumors, while *BRCA2* was generally retained (LOH: 3/19; loss: 4/19). *RAD51C* also displayed aberrations relatively frequently, with loss in eight tumors and LOH in 10 tumors. The mean HRD score was higher in cases displaying certain genetic alterations in *BRCA1*, *BRCA2*, and *RAD51C* as follows:

- *BRCA1* loss; mean HRD score 43 (loss) vs. 28 (no loss), (*p* = 0.048)
- *BRCA1* LOH; mean 42 (LOH) vs. 29 (no LOH), (*p* = 0.12)
- *BRCA2* loss; mean 50 vs. 34 (*p* = 0.18)
- *BRCA2* LOH; mean 58 vs. 34 (*p* = 0.0070)
- *RAD51C* loss; mean 51 vs. 28 (*p* = 0.0010)
- *RAD51C* LOH; mean 48 vs. 26 (*p* = 0.0021).

Additional genes commonly displaying LOH in this cohort (>10 tumors) included *RPA1*, *PIAS4*, *FANCA*, *CDK12*, *RAD51D*, and *FANCF*, and additional genes displaying genomic loss in >10 tumors included *PIAS4*, *FANCA*, *FANCF*, *TIPIN*, *SMC5*, *RMI1*, *PIAS1*, *FANCC*, and *ABL1.* Homozygous deletions in these genes were rare, with the *WRN* gene most frequently displaying deletions (found in three tumors; outlined in Supplementary Figure S1C and Supplementary Table S4). One case (E263) displayed homozygous deletions in nine genes, one of which was a core HRD gene, as well as several other Fanconi Anemia genes; copy-number plots also confirmed large deletions in all these regions.

In this cohort, the HRD phenotype did not influence survival, but both the size of the groups and small number of events are limiting factors. Thus, no firm conclusions regarding outcome in relation to HRD score in SEC can be drawn based on the present study (Supplementary Figure S2).

### 3.3. HRD and NHEJ-Associated Genes

There was no association between HRD score and genomic aberrations in NHEJ-associated genes, including LOH, genetic loss or homozygous deletions, in our cohort. Of note, LOH and/or genetic loss was fairly common in the genes encoding 53BP1, Ku70, XRCC4 and DNA-PKcs (aberrations in approximately 30–60% of tumors) but none of the tumors displayed homozygous deletions in any of the NHEJ-associated genes (Supplementary Figure S3).

### 3.4. Gene Amplifications

**Figure 3:**
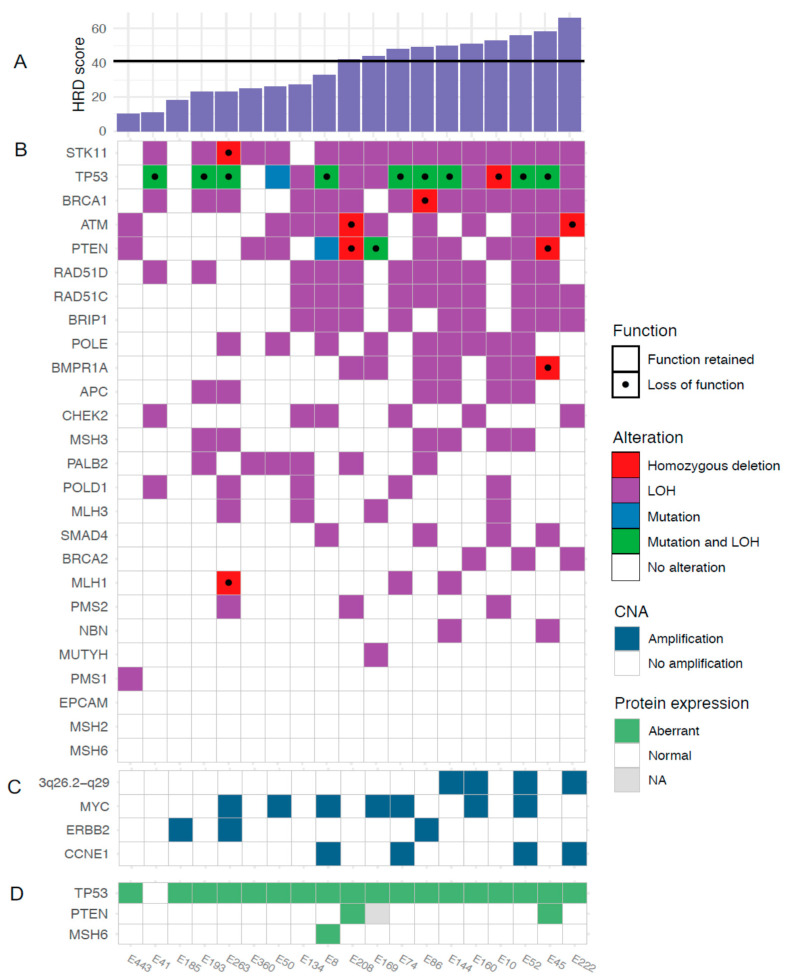
Somatic mutations and copy-number aberrations in relation to HRD score. (**A**) HRD scores, (**B**) genetic alterations, (**C**) amplifications, and (**D**) protein expression.

To further characterize the genomic features of SEC in relation to HRD, we analyzed the occurrence of oncogene amplifications. GISTIC analyses identified nine amplification peaks, three of which contained oncogenes previously reported in SEC by the TCGA (data available in Supplementary Table S5 and Supplementary Figure S4). The proto-oncogene *MYC* (8q24.21) was amplified in seven out of 19 tumors, the cell cycle regulator *CCNE1* (19q12) in four tumors and the growth factor receptor *ERBB2* (17q12) in three tumors (Figure 1A). Amplifications were not associated with HRD scores. Further, 16 out of 19 tumors harbored gains involving *MYC* (Figure 1B). Amplification of a wide region on chromosome 3 (3q26.2-q29), not reported by the TCGA in EC but described in EC as well as several other cancer forms by others was observed in four tumors and gain was observed in 17 tumors [30,31,32]. This region includes the potential driver genes *SOX2, ECT2, PRKCI,* and *PIK3CA*. The mean HRD score was higher in cases with amplification in this region compared to cases with no amplification (mean 56 (amplified) vs. 33 (non-amplified), *p* = 0.001; Figure 3C).

### 3.5. Targeted Sequencing

Known pathogenic somatic mutations were found in *TP53* (11 tumors, 58%) and *PTEN* (two tumors, 10.5%). Overall, the presence of a *TP53* mutation appeared unrelated to HRD score. The number of *PTEN* mutations were too few to allow any conclusions (Figure 3 and Appendix A). We also combined the copy-number and sequencing data for the 27 genes in the targeted sequencing panel. In addition to frequent *TP53* mutations, rare loss of function mutations were identified in *PTEN*, *ATM*, *STK11*, *BRCA1*, *BMPR1A* and *MLH1*. Figure 3B shows the genetic alterations in the sequenced genes across all tumors, sorted by HRD score.

### 3.6. Copy-Number Signatures

**Figure 4:**
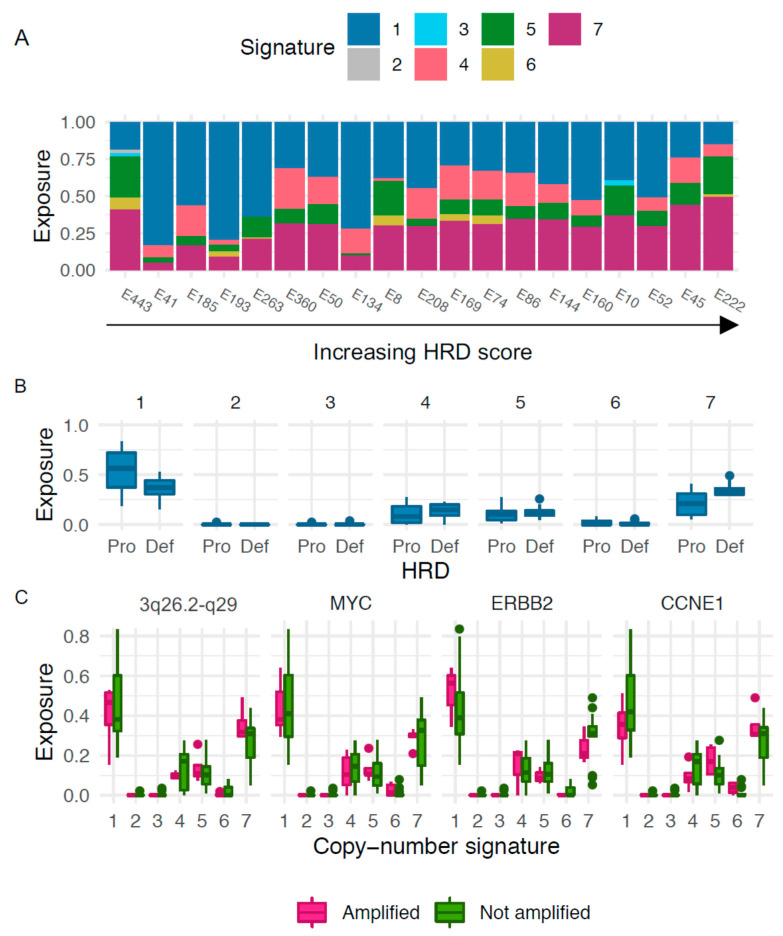
Copy-number signatures. (**A**) Distribution of exposure to the seven copy-number signatures with cases sorted by increasing HRD score. (**B**) Exposures to copy-number signatures for cases determined either HR proficient, “Pro” (score < 42), or HR deficient, “Def (score > 42). (**C**) Copy-number signatures in relation to amplified genes.

We next explored patterns of genomic aberrations in SEC using copy-number signatures previously described in HGSOC. The most predominant signatures in our cohort were copy-number signature 1, which, when analyzing the continuous HRD values, was negatively correlated with HRD score (r = −0.48, (−0.76; −0.026), *p* = 0.040), and copy-number signature 7, which was positively correlated with HRD score (r = 0.64, (0.27; 0.85), *p* = 0.0029; Figure 4A). Applying the previously described cut-off of >42, the HRD tumors were associated with signature 7 (mean exposure 0.35 vs. 0.22 for HRD vs. non-HRD, *p* = 0.013, Figure 4B). There was a weak association between the non-HRD tumors and signature 1 (mean exposure 0.37 vs. 0.53, *p* = 0.074, Figure 4B). No apparent association between outcome and copy-number signatures was observed, but the groups were too small for formal survival analyses (data not shown). We next investigated the connection between exposure to different copy-number signatures and amplification of the oncogenes previously discussed, suggesting a possible association between exposure to signature 1 and amplifications in *ERBB2* and *CCNE1*. However, small sample sizes precluded formal analyses (Figure 4C).

### 3.7. Immunohistochemistry

Of the 19 tumors in our cohort, 18 (95%) showed aberrant positive p53 staining and were considered *TP53* mutated, and two out of 18 (11%) showed loss of PTEN (data missing for one case). One case was regarded potentially MMR mutated, displaying loss of MSH6 protein.

Concordance between pathogenic mutations found through *TP53* sequencing and p53 IHC results was found for 10 out of 19 (53%) cases. Two of the discordant cases displayed mutations of unknown significance, likely causing the aberrant IHC staining. To explore the discordance further, copy-number data were interrogated with regard to regulators of the p53 protein. This revealed one case with amplification of *c-Myc* and loss of *MDM2*, regulators of p53 stability, and three with gain of *c-Myc*, but no amplification [33]. Detailed analyses are presented in Appendix A.

## 4. Discussion

SEC is an unusual but aggressive subtype of EC [2]. Comprising not only morphologic and histopathologic differences compared with the more frequent endometrioid EC, but also unique genomic features, SEC requires special attention and a new mindset with regard to refined therapeutics. The ProMisE classification, inspired by TCGA data, has established four molecular subtypes of EC and confirms the many similarities between SEC and HGSOC, such as frequent *TP53* mutations and copy-number alterations [3,6]. This encourages comparisons between these two tumor types in relation to approved treatment options. Since PARP inhibitors are active and result in substantial improvements in prognosis, especially among HR deficient HGSOCs, HRD and the potential benefit from PARP inhibitors are of great interest also in SEC. This is underlined by the efficacy of the PARP inhibitor olaparib presented in a recent case report of SEC [34]. To date, HRD has been shown to occur in about 50% of all HGSOCs, and HRD is to a large extent due to mutations in the *BRCA1* and *BRCA2* genes and these mutations are frequently in the germline [12,13,35]. HRD is also predictive of survival as well as response to PARP inhibitors and platinum in HGSOC [13,36,37]. Apart from the frequency of HRD, which has been reported to be comparable in HGSOC and SEC, and the reported occurrence of *RAD51* aberrations, the genomic landscape of HRD-associated genes as well as the treatment predictive value of HRD is still to be elucidated in SEC [17,18]. We therefore set out to genomically characterize a consecutive series of SECs using copy-number analyses, targeted sequencing and IHC used in routine diagnostics to investigate the presence and relevance of an HRD phenotype among SECs.

Using a previously defined cut-off for HRD (>42), we revealed that 53% of the SEC tumors in our cohort displayed an HRD phenotype [8]. This is in line with previous publications, although from smaller case series, demonstrating that HRD occurs only among non-endometrioid, *TP53* mutated ECs [18]. To our knowledge, the current study is the largest study of HRD-associated genomic events in a refined and well-annotated consecutive SEC cohort. We could also show that all three components of the HRD score contributed equally to the total score, i.e., the HRD score is as such likely to be a relevant measure of the HRD phenotype in SEC. Statistically significant associations between LOH and high HRD scores were also found. It is important to note that global LOH is a component of the total HRD score; hence, the correlation between a high HRD score and LOH in HRD genes may be a consequence of global LOH. It is however interesting that loss of *BRCA1* was found to be frequent in the current study and predominantly a feature of tumors with high HRD scores, indicating that loss of *BRCA1* could be an important contributor to the HRD phenotype. This is in line with a previous publication [15]. Likewise, frequent genetic loss and LOH was found in *RAD51C*, which is also a well-described DNA repair gene associated with *BRCA*-like features [35]. Hence, LOH in *BRCA1* or *RAD51C* may constitute the first hits underlying the development of HRD in SEC. Taken together, these findings further stress that SEC is truly a unique entity among ECs. Apart from the presence of gene amplification previously described to occur frequently in ECs, we also detected amplification of a wide region on chromosome 3 (3q26.2-29) which was associated with a high HRD score. This region involves potential driver genes such as *SOX2*, *ECT2*, *PRKC1*, and *PIK3C*. Amplification of this region has previously been reported in a wide range of cancers including EC [32]. Considering their prevalence in SEC and association to the HRD phenotype, these genes may be of interest for further investigation regarding targeted therapeutics. To take the investigation one step further, copy-number signatures were analyzed to investigate the genomic processes in SEC. The seven signatures used were derived from HGSOC, and four of these were of particular interest with respect to the current HRD and treatment focus; signature 3, corresponding to *BRCA1/2*-related HRD mechanisms; signature 7, corresponding to alternative, non-*BRCA*-related HRD mechanisms; signature 1, corresponding to RAS signaling and platinum resistance; and signature 6, corresponding to extremely high copy-number changes resulting in focal amplification, and correlation with age at diagnosis. Signature 1 is associated with a poor overall prognosis in HGSOC, whereas signatures 3 and 7 are associated with a favorable prognosis [22]. Although the current study had too immature follow-up data to draw any conclusions regarding outcome, it is interesting and reassuring to note that the most prominent signatures found in SEC were signatures 1 and 7, with signature 1 negatively correlated to HRD score and signature 7 positively correlated to HRD score. We did not find an enrichment of signature 3 in the present SEC cohort, despite the high frequency of HRD as well as LOH in *BRCA1*. This may indicate that although the HRD phenotype is frequent among SECs, it may not reflect defective DNA double-strand repair which is necessary for inducing PARP synthetic lethality. Furthermore, it stresses that although the HRD phenotype and possibly even genetic alterations in *BRCA1* are frequent in SEC, loss of function mutations in *BRCA1/2* and *BRCA*-related genes are rare. The mechanisms leading to the HRD phenotype may hence be different from those in HGSOC. This is in line with the findings from Ashely et al., whose mutational signatures (in comparison with the copy number-based signatures analyzed in the current study) indicated that HRD might be less functionally relevant in EC than could be expected [21]. This also aligns with reports showing that HGSOCs associated with non-*BRCA*-related HRD respond to PARP inhibition to a lesser extent than HGSOCs associated with *BRCA* mutations [38].

We detected mutations in *TP53* in 58% of our cases using targeted sequencing, which is slightly less than expected, but could probably be explained by normal cell contamination due to superficial tumors, warranting deeper sequencing depth [3]. However, aberrant p53 IHC staining was detected in 18 out of 19 of our tumor samples, and loss of PTEN staining, indicating loss of *PTEN* gene function, was detected in two cases, in line with previous studies [39]. The location of the specific mutations in relation to the antibody’s binding site may confound the IHC interpretation, potentially explaining the cases where either *TP53* or *PTEN* missense mutations were revealed, but no aberrant IHC staining was detected. For cases where mutations were inferred by IHC alone, the mutation may have been missed or filtered out from the sequencing data due to unknown significance or insufficient number of reads, or alternatively the protein expression pattern may be the result of epigenetic mechanisms. For *TP53* in particular, a disruption in the regulation of *TP53* via alterations in, e.g., *MDM2*, *p14/ARF*, or *c-Myc* may also be present. Amplification of *c-Myc*/loss of *MDM2* was found in one of our cases without a detectable *TP53* mutation. These genomic alterations may cause deregulation of p53 protein turnover, hence resulting in an aberrant IHC readout [33]. Likewise, homozygous deletions were found in the two cases with loss of PTEN protein expression that did not harbor mutations detectable by sequencing.

We also detected one case with a potential *MSH6* mutation, but the MMR mutation frequency in EC in general is approximately 15–20% [5,40]. This may be of importance for future studies since HGSOC in vitro experiments have shown improved response to PARP inhibition in cases with co-occurrence of HRD and mutations in either MMR or NER genes [14]. Likewise, other HGSOC in vitro experiments postulate that the error-prone DNA double-strand repair mechanism NHEJ is aberrantly activated by PARP inhibitors in HR deficient cells, leading to increased genomic instability. Conversely, deficient NHEJ would then imply decreased PARP inhibitor effect in HR deficient cells [27]. Since PARP inhibition in HGSOC results in prolonged survival, a decreased effect due to NHEJ deficiency would be prognostically unfavorable. In line with this, a previous publication has shown that NHEJ proficient ECs with loss of *RAD51* have an increased disease-free survival compared to those without *RAD51* loss [41]. In the current study, HRD scores were not related to aberrations in any of the core NHEJ genes. The low incidence of deaths and the limited size of the cohort do not allow any conclusions regarding the effect of NHEJ alterations on survival.

To summarize, we show that the genomic scars associated with the HRD phenotype occur frequently in SEC, but in general SECs appear to harbor less diverse copy-number alteration patterns, likely reflecting the involvement of fewer mutational processes than may be inferred from HGSOC data [22]. Somatic mutations in *BRCA*/HRD-associated genes are also infrequent in SEC, and do not appear to explain the HRD phenotype in these cancers. However, copy-number signatures revealed an association between tumors with an HRD phenotype and the non-*BRCA*-related HRD signature from HGSOC, implying that genes other than *BRCA1/2* as well as combinations of genomic gains/losses in HRD genes warrant further investigation to uncover the mechanisms underlying the HRD phenotype in SEC. Although the current cohort is limited in size, it is large in its context and well-characterized using different techniques. Thus, this study emphasizes that SEC is not identical to HGSOC but is a tumor entity of its own. A subset of SEC patients may still potentially benefit from PARP inhibition, but the proportion of patients, the extent of potential treatment benefit, as well as the exact underlying mechanism remain to be elucidated.

## 5. Conclusions

SECs frequently display phenotypic signs of HRD (HRD phenotype), and harbor frequent copy-number alterations, but few mutations in HRD-associated genes. The HRD phenotype is mostly non-*BRCA* related, but more information is needed to uncover the precise mechanisms behind, and relevance of this phenotype with regards to, e.g., targeted therapies.

## Figures and Tables

**Table 1:** Summary of patient-related and clinical data.

Variable	HR Deficient * Subgroup	HR Proficient Subgroup
Number of patients (%)	10 (53)	9 (47)
Median HRD score (range)	50.5 (42–66)	23 (10–33)
Median age at diagnosis (range)	76 (69–90)	72 (56–90)
FIGO stage (%)		
I	6 (60)	6 (67)
II	0	0
III	3 (30)	1 (11)
IV	1 (10)	2 (22)
Median follow-up, months (range)	45.5 (2–60)	48 (29–60)
Number deceased (%)	2 (20)	3 (33)

* HR deficiency defined as HRD score > 42.

## Data Availability

The data presented in this study are available on request from the corresponding author. The data are not publicly available due to GDPR restrictions.

## Supplementary Materials

The following are available online at https://www.mdpi.com/2072-6694/13/2/254/s1, Figure S1: Heatmap of cases and genes with genetic loss, LOH, and homozygous deletions, Figure S2: Kaplan-Meier estimate of overall survival (OS) for HR deficient vs. proficient cases, Figure S3: Heatmap of cases and genes with genetic loss, LOH, and homozygous deletions in NHEJ-associated genes, Figure S4: Significant amplification (A) and deletion (B) peaks form the GISTIC analysis, Table S1: Detailed clinical data, Table S2: Variants detected through targeted sequencing, Table S3: Overview of antibodies used in the study, Table S4: Genes in deletion peaks from GISTIC, Table S5: Genes in amplification peaks from GISTIC.

## Abbreviations

BER	Base Excision Repair
CI	Confidence Interval
CNA	Copy-Number Alteration
EC	Endometrial cancer
FGA	Fraction of Genome Altered
FIGO	International Federation of Gynecology and Obstetrics
FFPE	Formalin-Fixed Paraffin-Embedded
GISTIC	Genomic Identification of Significant Targets in Cancer
H&E	Hematoxylin & Eosin
HGSOC	High-Grade Serous Ovarian Cancer
HR	Homologous Recombination Repair
HRD	Homologous Recombination Repair Deficiency
IHC	Immunohistochemistry
LOH	Loss of Heterozygosity
LST	Large-Scale State Transitions
MMR	Mismatch Repair
NER	Nucleotide Excision Repair
NHEJ	Non-Homologous End Joining
PARP	Poly (ADP-Ribose) Polymerase
SEC	Serous Endometrial Cancer
SI	Supplementary Information
SNP	Single Nucleotide Polymorphism
TAI	Telomeric Allelic Imbalance

## Appendix A

### Apendix A. 1. Methods

#### Apendix A. 1. 1. Targeted Sequencing

Sequencing was performed on both tumor tissue and whole blood in order to filter out germline variants. DNA extraction from frozen tissue was performed using the All-prep mini kit (Qiagen, Venlo, The Netherlands) and DNA extraction from whole blood was performed using kit CMG-1497 (Perkin Elmer, Germany), on a Chemagic Prime (PerkinElmer chemagen Technologie GmbH, Germany).

Library preparation was performed using the KAPA HyperPrep Kit (Roche Diagnostics AB^®^, Basel, Switzerland) with an input of 250 ng DNA. The Twist bioscience protocol was used for target enrichment of the DNA library for the predetermined gene panel. Targeted sequencing was performed using the NextSeq 500/550 Hi Output KT v2.5 (300CYS) (Illumina^®^, San Diego, CA, USA) with read lengths of 2 × 151 and 2 × 8 bp index reads. Demultiplexing was performed using PICARD (v. 2.20.8), alignment using NovoAlign (Novocraft, v. 4.0). Freebayes v. 5 was used for variant calling, and XHMM (v. 1.0; -L custom target boundaries), Manta (v. 1.2.1) and Melt (v. 2.1.3), for discovery of structural variants. The mean bait coverage for each sample ranged from 449–2080 reads (mean 931). For the variant calling the min-alternate-fraction threshold was 3%, and min-alternate-count was 3 reads. Annotations were extracted from Annovar (17 June 2015, database: 150623).

Filtration of variants detected was performed in several steps using Freebayes v.5, GATK VariantAnnotator v. 3.4-46 and bcftools v. 1.2. Variants were flagged if they displayed any of the following quality discrepancies:

QUAL < 10

DP < 5

SAF < 2 | SAR < 2 *

RPL < 2 | RPR < 2 **

AB < 0.05 & AB > 0

(TYPE = “snp” & FS > 60) | (TYPE ! = “snp” & FS > 200)

*SAF/SAR—Number of alternate observations on the forward/reverse strand

**RPR/RPL—Reads Placed Right/Left: number of reads supporting the alternate balanced to the right (3’) (or left, 5’) of the alternate allele.

Finally, a manual assessment and subsequent filtration of PCR artefacts was performed.

#### Apendix A. 1. 2. Gene Amplifications and Deletions

Gains and losses were defined as each segment with a total copy-number of at least ploidy +0.6, or ploidy −0.6, respectively. Amplifications were defined as 2× ploidy and homozygous deletions as a total copy-number of 0. In cases where a gene overlapped two segments, the segment containing the largest part of the gene was used. For the GISTIC analysis default was used for all settings.

### Apendix A. 2. Extended Results

#### Targeted Sequencing and Immunohistochemistry

Aberrant p53 protein expression was found in 18/19 (95%) cases using immunohistochemistry (IHC). Known pathogenic mutations in *TP53* were found in 11/19 cases (58%) using targeted sequencing, of which 10 were concordant with the IHC results. Possible explanations for the discordance are:

- For two cases displaying aberrant p53 overexpression without corresponding *TP53* mutation, mutations of unknown significance, likely causing the aberrant IHC staining, were found
- For two cases displaying aberrant p53 overexpression without corresponding *TP53* mutation, mutations were detected but were filtered out due to poor quality, likely because of high normal cell contamination
- For one case displaying aberrant p53 overexpression without corresponding *TP53* mutation, amplification of *c-Myc* and loss of *MDM2*, regulators of p53 stability, were found
- For three cases displaying aberrant p53 overexpression without corresponding *TP53* mutation, gain of *c-Myc* was found
- For one case, not regarded as p53 aberrant by IHC, a missense mutation was found which may confound IHC interpretation

Loss of PTEN protein expression was found in 2/18 (11%) cases (data missing for one case). Known pathogenic mutations in *PTEN* were found in 2/19 (10.5%) cases. These cases were not concordant. Possible explanations are:

- For one case with0020a missense *PTEN* mutation but retained protein expression in IHC, no LOH in the gene was detected which means that gene function may be retained
- For one case with a missense *PTEN* mutation data on protein expression was missing
- For two cases with loss of PTEN protein staining by IHC but no corresponding mutations, homozygous deletions in this region were found which may affect protein expression
