# Supplementary material for: Homologous Recombination Repair Mechanisms in Serous Endometrial Cancer
Source: Cancers (Basel). 2021 Jan 12;13(2):254. doi: 10.3390/cancers13020254 (PMC7827019; doi:10.3390/cancers13020254)
Supplement: Supplementary file 1 [file cancers-13-00254-s001.zip › cancers-1045961-supplementary R1 - Copy/Supplementary Figure S1.pdf]

Supplementary Figure S1.
Heatmap of cases and genes with genetic loss (A), LOH (B), and homozygous deletions (C), respectively in HRD-associated genes. Bar plots show summarized data on each axis. Genes defined as "core HRD genes" are shown in bold.

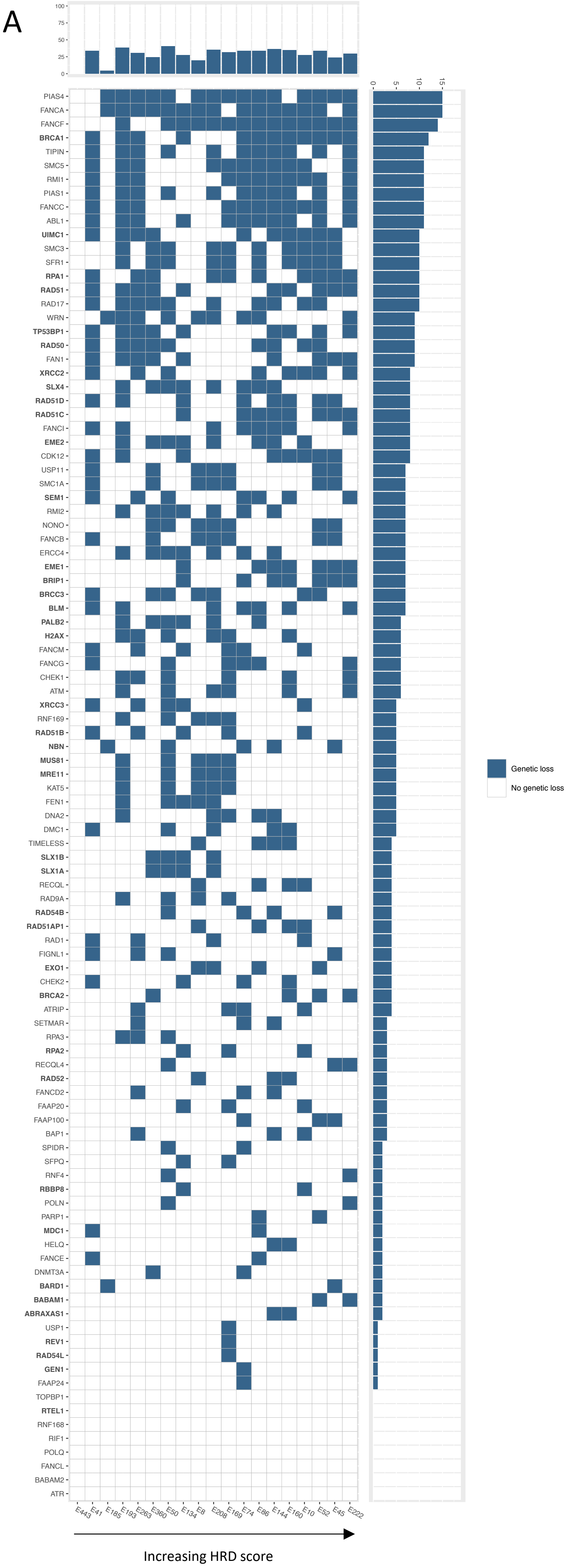

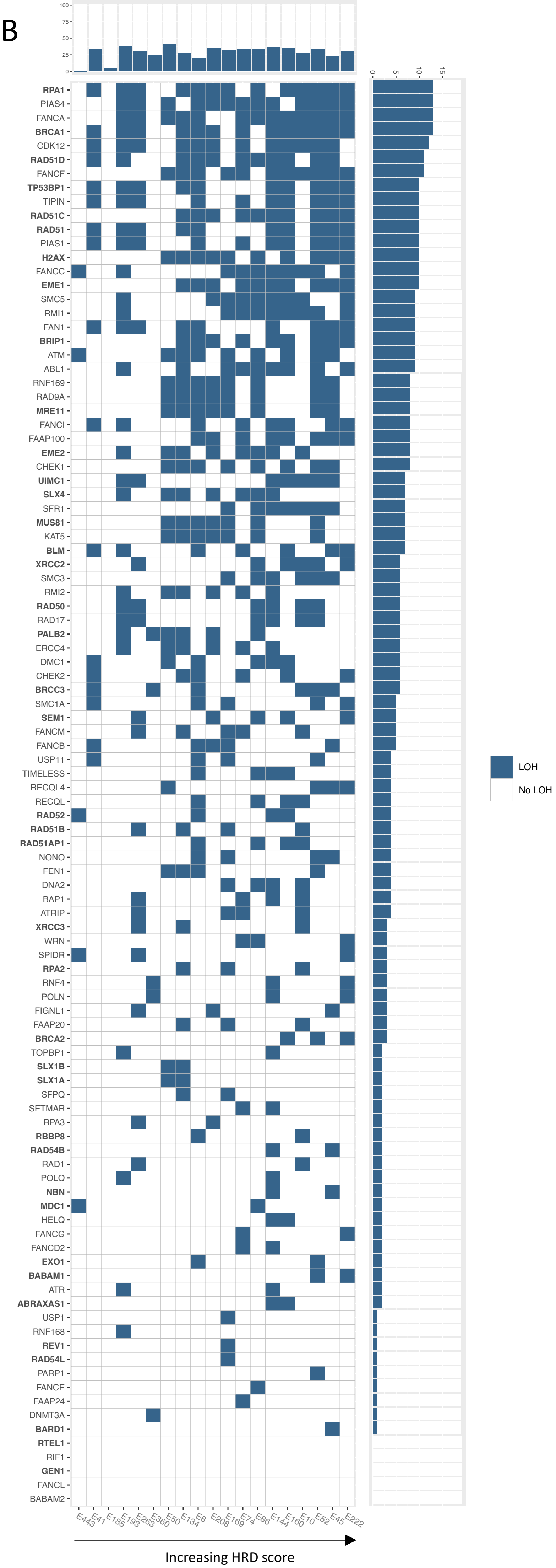

B
100
75
50
25
0
0
5
10
15
RPA1
PIAS4
FANCA
BRCA1
CDK12
RAD51D
FANCF
TP53BP1
TIPIN
RAD51C
RAD51
PIAS1
H2AX
FANCC
EME1
SMC5
RMI1
FAN1
BRIP1
ATM
ABL1
RNF169
RAD9A
MRE11
FANCI
FAAP100
EME2
CHEK1
UIMC1
SLX4
SFR1
MUS81
KAT5
BLM
XRCC2
SMC3
RMI2
RAD50
RAD17
PALB2
ERCC4
DMC1
CHEK2
BRCC3
SMC1A
SEM1
FANCM
FANCB
USP11
TIMELESS
RECQL4
RECQL
RAD52
RAD51B
RAD51AP1
NONO
FEN1
DNA2
BAP1
ATRIP
XRCC3
WRN
SPIDR
RPA2
RNF4
POLN
FIGNL1
FAAP20
BRCA2
TOPBP1
SLX1B
SLX1A
SFPQ
SETMAR
RPA3
RBBP8
RAD54B
RAD1
POLQ
NBN
MDC1
HELQ
FANCG
FANCD2
EXO1
BABAM1
ATR
ABRAXAS1
USP1
RNF168
REV1
RAD54L
PARP1
FANCE
FAAP24
DNMT3A
BARD1
RTEL1
RIF1
GEN1
FANCL
BABAM2
E443
E41
E185
E193
E263
E360
E50
E134
E8
E208
E169
E74
E86
E144
E160
E10
E52
E45
E222
Increasing HRD score
LOH
No LOH

C

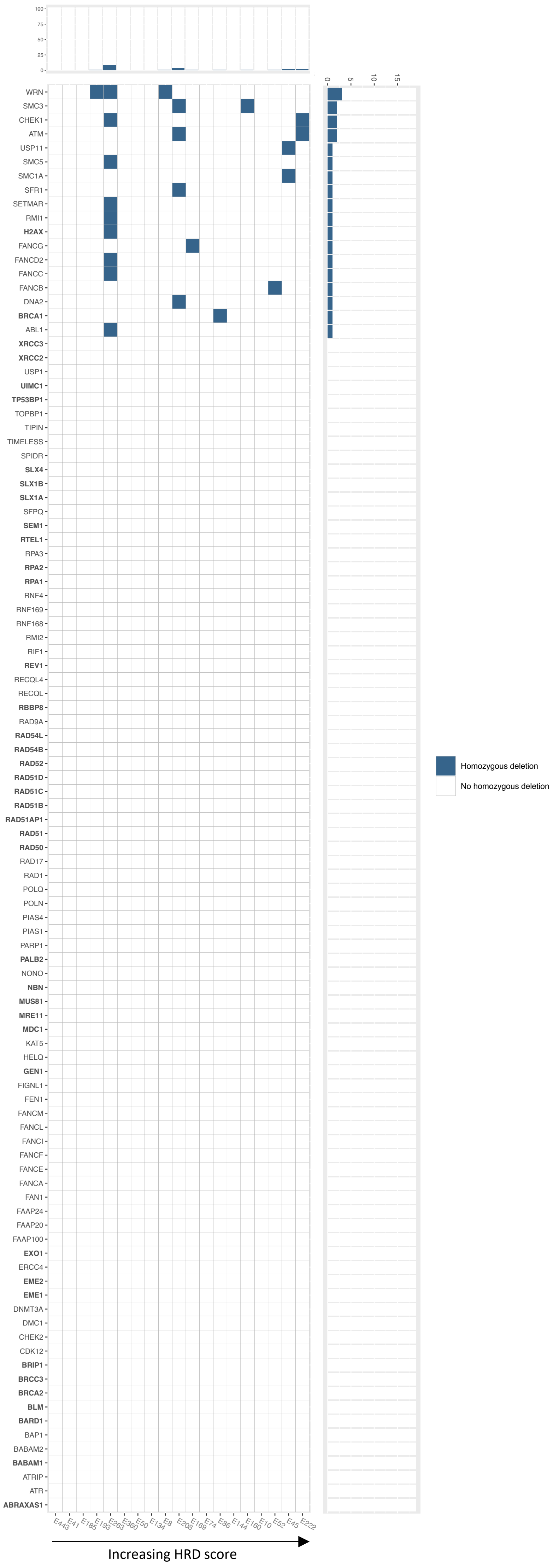
100
75
50
25
0
0
5
10
15
WRN
SMC3
CHEK1
ATM
USP11
SMC5
SMC1A
SFR1
SETMAR
RMI1
H2AX
FANCG
FANCD2
FANCC
FANCB
DNA2
BRCA1
ABL1
XRCC3
XRCC2
USP1
UIMC1
TP53BP1
TOPBP1
TIPIN
TIMELESS
SPIDR
SLX4
SLX1B
SLX1A
SFPQ
SEM1
RTEL1
RPA3
RPA2
RPA1
RNF4
RNF169
RNF168
RMI2
RIF1
REV1
RECQL4
RECQL
RBBP8
RAD9A
RAD54L
RAD54B
RAD52
RAD51D
RAD51C
RAD51B
RAD51AP1
RAD51
RAD50
RAD17
RAD1
POLQ
POLN
PIAS4
PIAS1
PARP1
PALB2
NONO
NBN
MUS81
MRE11
MDC1
KAT5
HELQ
GEN1
FIGNL1
FEN1
FANCM
FANCL
FANCI
FANCF
FANCE
FANCA
FAN1
FAAP24
FAAP20
FAAP100
EXO1
ERCC4
EME2
EME1
DNMT3A
DMC1
CHEK2
CDK12
BRIP1
BRCC3
BRCA2
BLM
BARD1
BAP1
BABAM2
BABAM1
ATRIP
ATR
ABRAXAS1
E443
E41
E185
E193
E263
E360
E50
E134
E8
E208
E169
E74
E86
E144
E180
E10
E52
E45
E222
Increasing HRD score
Homozygous deletion
No homozygous deletion
