# Supplementary material for: Homologous Recombination Repair Mechanisms in Serous Endometrial Cancer
Source: Cancers (Basel). 2021 Jan 12;13(2):254. doi: 10.3390/cancers13020254 (PMC7827019; doi:10.3390/cancers13020254)

**Supplementary Figure S2.** Kaplan-Meier estimate of overall survival (OS) for HR deficient *vs.* proficient cases. Each descending step in the plot marks a deceased patient and vertical tick-marks represent patients still alive at last follow-up.

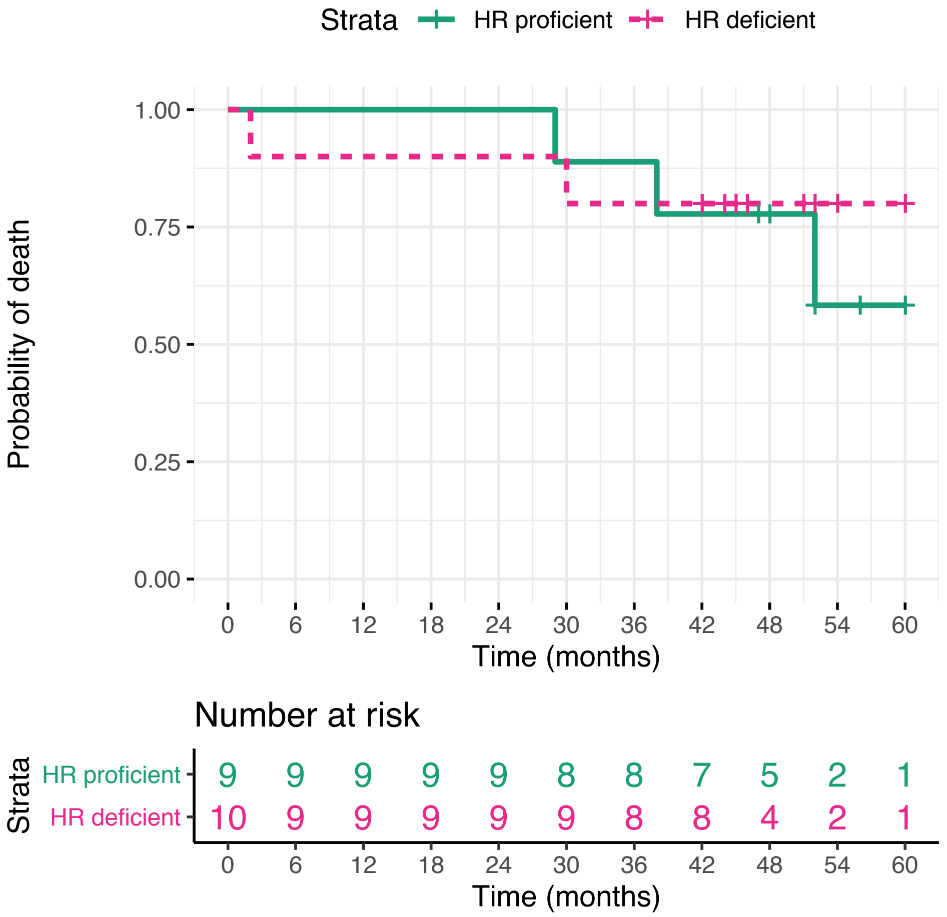

Supplement: Supplementary file 1 [file cancers-13-00254-s001.zip › cancers-1045961-supplementary R1 - Copy/Supplementary Figure S2.pdf]
