# Supplementary material for: Homologous Recombination Repair Mechanisms in Serous Endometrial Cancer
Source: Cancers (Basel). 2021 Jan 12;13(2):254. doi: 10.3390/cancers13020254 (PMC7827019; doi:10.3390/cancers13020254)
Supplement: Supplementary file 1 [file cancers-13-00254-s001.zip › cancers-1045961-supplementary R1 - Copy/Supplementary Figure S3.pdf]

Supplementary Figure S3. Heatmap of cases and genes with genetic loss, LOH, and homozygous deletions in NHEJ-associated genes

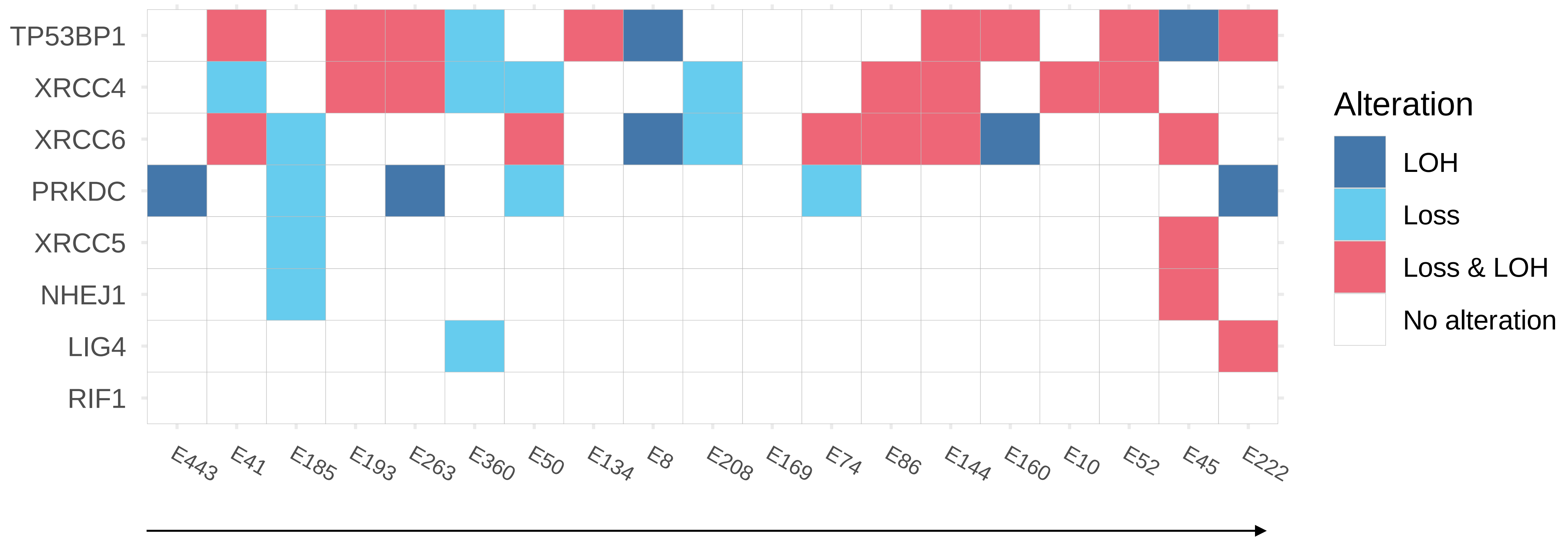

Increasing HRD score
