# Supplementary material for: Homologous Recombination Repair Mechanisms in Serous Endometrial Cancer
Source: Cancers (Basel). 2021 Jan 12;13(2):254. doi: 10.3390/cancers13020254 (PMC7827019; doi:10.3390/cancers13020254)
Supplement: Supplementary file 1 [file cancers-13-00254-s001.zip › cancers-1045961-supplementary R1 - Copy/Supplementary Figure S4.pdf]

Supplementary Figure S4. Significant amplification (A) and deletion (B) peaks from the GISTIC analysis.

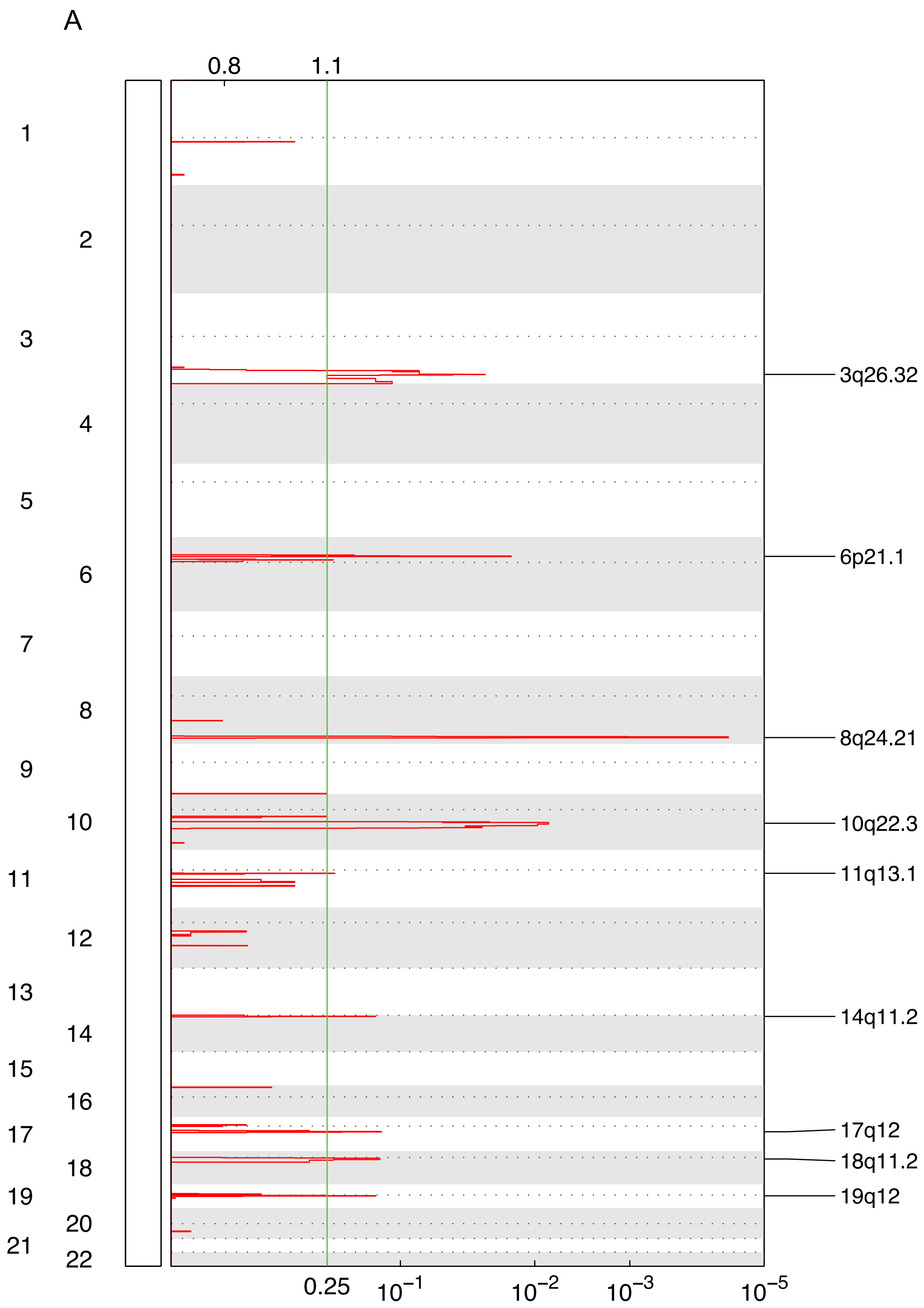

B

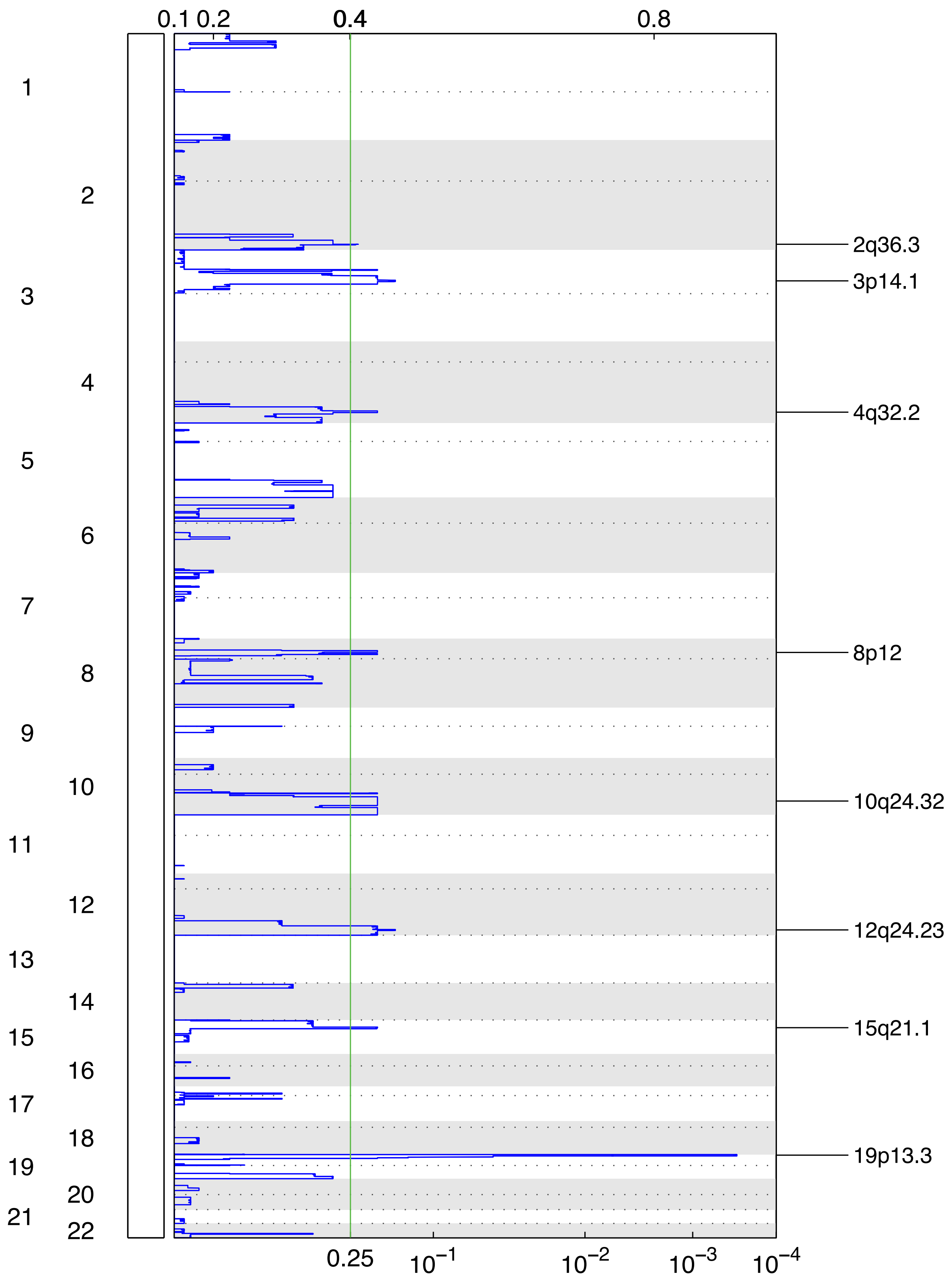
0.1 0.2
0.4
0.8
1
2
3
4
5
6
7
8
9
10
11
12
13
14
15
16
17
18
19
20
21
22
2q36.3
3p14.1
4q32.2
8p12
10q24.32
12q24.23
15q21.1
19p13.3
0.25
10^{-1}
10^{-2}
10^{-3}
10^{-4}
