# Supplementary material for: Homologous Recombination Repair Mechanisms in Serous Endometrial Cancer
Source: Cancers (Basel). 2021 Jan 12;13(2):254. doi: 10.3390/cancers13020254 (PMC7827019; doi:10.3390/cancers13020254)
Supplement: Supplementary file 1 [file cancers-13-00254-s001.zip › cancers-1045961-supplementary R1 - Copy/Supplementary Table S1.pdf]

Supplementary Table S1

Detailed clinical data, sorted from the highest HRD score to the lowest. Tumors with HRD score ≥42 display an HRD phenotype.

Id nr	HRD score	Stage	Age	p53*	MMR**	PTEN***	Heredity	Other information	Adjuvant treatment	Relapse site
222	66	IIIC1	69	1	N/A	1			P/C¥ x 4	
45	58	IA	83	1	0	0		synchronous chronic lymphatic leukemia	No†	
52	56	IIIC1	81	1	0	1			P/C¥ x 4	
10	53	IA	90	1	0	1			No†	
160	51	IVB	72	1	0	1	father colon cancer<br>brother prostate cancer		P/C¥ x 6	ascites
144	50	IA	73	1	0	1			P/C¥ x 4	
86	49	IB	79	1	0	1			P/C¥ x 4	
74	48	IIIC1	80	1	0	1	father head & neck cancer<br>brother head & neck cancer<br>sister breast cancer		P/C¥ x1	
169	44	IB	72	1	0	N/A			P/C¥ x 4	
208	42	IA	71	1	0	0	mother unspecified abdominal cancer		P/C¥ x 4	
8	33	IA	90	1	1	1			No†	
134	27	IA	74	1	0	1	aunt endometrial cancer		P/C¥ x 4	
50	26	IB	81	1	0	1			C‡ x 4	
360	25	IA	64	1	0	1			P/C¥ x 4	
193	23	IVB	72	1	0	1			P/C¥ x 6	
263	23	IA	68	1	0	1			P/C¥ x 4	

Supplementary Table S1, continued

185	18	IVB	71	1	0	1			P/C¥ x 5	ascites<br>pleural effusion
41	11	IA	82	0	0	1			No†	
443	10	IIIC2	56	1	0	1	mother unspecified cancer		P/C¥ x 4	ascites

*p53 IHC is dichotomized as 0 = wild type, and 1 = p53 mutation

**MMR IHC is a dichotomized sum where 1 = mutation

***PTEN IHC is dichotomized as 0 = loss of function, and 1 = retained function

†The reason for not administering adjuvant treatment was co-morbidity

‡C = carboplatin AUC6 x number of treatment cycles

¥paclitaxel 175 mg/m^2 and carboplatin AUC5 x number of treatment cycles

N/A = data missing
