# Supplementary material for: Homologous Recombination Repair Mechanisms in Serous Endometrial Cancer
Source: Cancers (Basel). 2021 Jan 12;13(2):254. doi: 10.3390/cancers13020254 (PMC7827019; doi:10.3390/cancers13020254)
Supplement: Supplementary file 1 [file cancers-13-00254-s001.zip › cancers-1045961-supplementary R1 - Copy/Supplementary Table S2.pdf]

Supplementary Table S2

Variants detected through targeted sequencing. All potentially pathogenic variants presented.

CHROM	POS	SAMPLE	REF	ALT	GENE	TOP_EFFECT	MEAN_AD	MEAN_AB	CLINVAR_classification	HGVS
10	89624296	E169	G	C	PTEN	nonsynonymous_SNV	153/581	0,792	pathogenic	c.70G>C
10	89692905	E8	G	A	PTEN	nonsynonymous_SNV	924/127	0,121	pathogenic	c.390G>A
10	89711900	E8	G	A	PTEN	nonsynonymous_SNV	955/109	0,102	pathogenic	c.518G>A
10	89720647	E193	-	G	PTEN	near_splice	102/68	0,4	unknown	c.802-4_804delinsA
12	133215703	E160	G	C	POLE	near_splice	434/74	0,146	unknown	c.5552+8C>G
16	23646295	E134	T	C	PALB2	synonymous_SNV	570/775	0,576	unknown	c.1572A>G
17	7577094	E74	G	A	TP53	nonsynonymous_SNV	153/227	0,597	pathogenic	c.844C>T
17	7577097	E193	C	T	TP53	nonsynonymous_SNV	157/456	0,744	pathogenic	c.841G>A
17	7577120	E144	C	A	TP53	nonsynonymous_SNV	40/424	0,914	pathogenic	c.818G>T
17	7577120	E52	C	T	TP53	nonsynonymous_SNV	174/333	0,657	pathogenic	c.818G>T
17	7577538	E45	C	T	TP53	nonsynonymous_SNV	389/120	0,236	pathogenic	c.743G>A
17	7577538	E41	C	T	TP53	nonsynonymous_SNV	152/778	0,837	pathogenic	c.743G>A
17	7577539	E8	G	A	TP53	nonsynonymous_SNV	717/123	0,146	pathogenic	c.742C>T
17	7577541	E169	-	*	TP53	nonframeshift_insertion	27/181	0,87	unknown	NA
17	7578177	E134	C	T	TP53	synonymous_SNV	1253/166	0,117	unknown	c.672G>A
17	7578203	E86	C	T	TP53	nonsynonymous_SNV	163/506	0,756	pathogenic	c.646G>A
17	7578271	E10	T	C	TP53	nonsynonymous_SNV	91/393	0,812	pathogenic	c.578A>G
17	7578406	E263	C	T	TP53	nonsynonymous_SNV	101/346	0,774	pathogenic	c.524G>A
17	7578556	E50	T	C	TP53	splicing	520/249	0,324	pathogenic	c.376-2A>G
17	7579377	E50	G	-	TP53	frameshift_deletion	448/163	0,267	pathogenic	c.310del

*TCATGCCGCCCATGCAGGAACTGTTACACA
