# Supplementary material for: Homologous Recombination Repair Mechanisms in Serous Endometrial Cancer
Source: Cancers (Basel). 2021 Jan 12;13(2):254. doi: 10.3390/cancers13020254 (PMC7827019; doi:10.3390/cancers13020254)
Supplement: Supplementary file 1 [file cancers-13-00254-s001.zip › cancers-1045961-supplementary R1 - Copy/Supplementary Table S3.pdf]

Supplementary Table S3

Overview of antibodies used in the current study

All sections were stained using BenchMark Ultra (Roche©) and the respecive antibody buffers.

For antibody detection either OptiView DAB or Ultra View Universal DAB (Roche©) was used.

Antibody	Vendor	Type	Clone	Dilution	Evaluation
**p53**	DAKO©	Mouse monoclonal	DO-7	1:100	1 = aberrant negative or >80% cells with aberrant positive nuclear staining*<br>0 = wild type (between ab. pos. and neg.)
**p16**	Ventana©	Mouse monoclonal	E6H4	RTU	1 = block positive (moderate-strong nucl. and cytoplasm. staining in 10-20 close cells*<br>0 = no staining or mosaic pattern
**ER**	Ventana©	Rabbit monoclonal	SP1	RTU	2 = ≥50% cells with nuclear staining*<br>1 = 10-49% cells with nuclear staining*<br>0 = <10% cells with nuclear staining
**PgR**	Ventana©	Rabbit monoclonal	100	RTU	2 = ≥50% cells with nuclear staining*<br>1 = 10-49% cells with nuclear staining*<br>0 = <10% cells with nuclear staining
**Ki67**	DAKO©	Mouse monoclonal	MIB-1	1:50	3 = ≥75% cells with nuclear staining in 200 nuclei in hot-spot areas<br>2 = 50-74% cells with nuclear staining in 200 nuclei in hot-spot areas<br>1 = 25-49 cells with nuclear staining in 200 nuclei in hot-spot areas<br>0 = <25% cells with nuclear staining in 200 nuclei in hot-spot areas
**WT1**	DAKO©	Mouse monoclonal	6F-H2	RTU	1 = ≥10% cells with nuclear staining*<br>0 = <10% cells with nuclear staining
**Napsin A**	Leica©	Mouse monoclonal	IP64	1:20	1 = diffuse, spotted cytoplasmic staining*<br>0 = other staining pattern than 1
**MLH1**	Leica©	Mouse monoclonal	ES05	1:100	1 = loss of staining**<br>0 = retained staining
**MSH2**	AH-Diagnostics©	Mouse monoclonal	G219-1129	1:100	1 = loss of staining**<br>0 = retained staining
**MSH6**	Biosite©	Rabbit monoclonal	EP49	1:100	1 = loss of staining**<br>0 = retained staining

Supplementary Table S4 continued

Antibody	Vendor	Type	Clone	Dilution	Evaluation
**PMS2**	Biosite©	Rabbit monoclonal	EP51	1:100	1 = loss of staining**<br>0 = retained staining
**PTEN**	Ventana©	Rabbit monoclonal	SP218	RTU	1 = diffuse cytoplasmic staining in >90% cells<br>0 = diffuse cytoplasmic staining in <1% cells***<br>heterogeneous = between 0 and 1

RTU = ready to use; 1-3=positive scoring, 0=negative scoring

*indicates mutation or overexpression; **MMR mutation; ***mutation/loss of function
