# Supplementary material for: Homologous Recombination Repair Mechanisms in Serous Endometrial Cancer
Source: Cancers (Basel). 2021 Jan 12;13(2):254. doi: 10.3390/cancers13020254 (PMC7827019; doi:10.3390/cancers13020254)
Supplement: Supplementary file 1 [file cancers-13-00254-s001.zip › cancers-1045961-supplementary R1 - Copy/Supplementary Table S4.pdf]

Supplementary Table S4 Genes included in significant GISTIC deletion peaks

cytoband	19p13.3	3p14.1	12q24.23	4q32.2
q value	0,00031532	0,15763	0,15898	0,18973
residual q value	0,00031532	0,15763	0,15763	0,18973
wide peak boundaries	chr19:1-2451267	chr3:52861770-70011694	chr12:100419710-133275309	chr4:149270911-190214555
genes in wide peak	AMH	ARF4	ACACB	hsa-mir-1979
	ATP5D	ERC2-IT1	ACADS	AGA
	AZU1	CACNA1D	ALDH2	SLC25A4
	HCN2	DNASE1L3	ARL1	ANXA2P1
	BSG	FHIT	ASCL1	CASP3
	CDC34	FLNB	ATP2A2	LRBA
	CIRBP	MITF	BCL7A	CLCN3
	CNN2	PDHB	SCARB1	CPE
	CSNK1G2	PRKCD	CMKLR1	CTSO
	CFD	PTPRG	COX6A1	DCTD
	ARID3A	ATXN7	CRY1	ETFDH
	EFNA2	TKT	DAO	F11
	ELANE	TMF1	DTX1	ACSL1
	GAMT	WNT5A	EIF2B1	FAT1
	MKNK2	SLMAP	STX2	FGA
	GPX4	ACOX2	GOLGA3	FGB
	GZMM	CADPS	GTF2H3	FGG
	OAZ1	SUCLG2	HPD	FRG1
	PALM	HESX1	IGF1	GK3P
	POLR2E	UBA3	MMP17	GLRB
	POLRMT	MAGI1	MSI1	GPM6A
	PRTN3	PSMD6	MVK	GRIA2
	PTBP1	ARL6IP5	MYBPC1	GUCY1A3
	RPS15	RPP14	MYL2	GUCY1B3
	STK11	FAM107A	NFYB	HMGB2
	TCF3	FRMD4B	NOS1	HPGD
	MADCAM1	FAM208A	OAS1	ING2

cytoband	19p13.3	3p14.1	12q24.23	4q32.2
genes in wide peak	SF3A2	LRIG1	OAS2	IRF2
	PLPP2	ERC2	OAS3	KLKB1
	AP3D1	APPL1	P2RX4	MTNR1A
	MED16	ARHGEF3	P2RX7	NEK1
	FSTL3	SFMBT1	PEBP1	NPY1R
	APC2	IL17RD	PAH	NPY2R
	ABCA7	PXK	PLA2G1B	NPY5R
	UQCR11	FEZF2	PMCH	GATB
	SBNO2	CHDH	POLE	PLRG1
	ARHGAP45	IL17RB	PPP1CC	PPID
	SHC2	CACNA2D3	PRKAB1	RPS3A
	TIMM13	DCP1A	PSMD9	MSMO1
	DAZAP1	LMOD3	PTPN11	SFRP2
	RNU6-1	ADAMTS9	PXMP2	TDO2
	FGF22	ABHD6	PXN	TLL1
	THEG	LRTM1	RAN	TLR2
	LSM7	C3orf14	RFC5	TLR3
	MBD3	SELK	RFX4	VEGFC
	MIER2	THOC7	RPL6	GLRA3
	PCSK4	KBTBD8	RPLP0	SORBS2
	C19orf24	RFT1	CLIP1	SAP30
	PLEKHJ1	ACTR8	ATXN2	SNORD73A
	BTBD2	SLC25A26	SELPLG	LRAT
	RNF126	FAM3D	SFSWAP	HAND2
	SPPL2B	C3orf49	TBX5	RAPGEF2
	WDR18	SNTN	TBX3	MFAP3L
	REXO1	SYNPR	HNF1A	MAB21L2
	PLPPR3	ASB14	TDG	ADAM29
	OR4F17	FAM19A4	HSP90B1	ANXA10
	ABHD17A	PRICKLE2	TXNRD1	KLHL2
	KLF16	C3orf67	UBC	SCRG1
	DOT1L	KCTD6	UNG	PALLD

cytoband	19p13.3	3p14.1	12q24.23	4q32.2
genes in wide peak	KISS1R	DNAH12	ZNF10	KIAA0922
	MUM1	PDE12	ZNF26	TRIM2
	MIDN	DENND6A	ZNF84	ANP32C
	R3HDM4	EOGT	ZNF140	FAM149A
	TMEM259	CCDC66	CDK2AP1	FBXO8
	TPGS1	LINC00698	BRAP	ARFIP1
	REEP6	SPATA12	ULK1	PDLIM3
	IZUMO4	FAM19A1	RASAL1	SPOCK3
	SCAMP4	ESRG	MAPKAPK5	AADAT
	ADAT3	LINC00994	DENR	FAM198B
	GRIN3B	MIR3136	OASL	ASIC5
	JSRP1	MIR4272	DYNLL1	GALNT7
	MOB3A	MIR3938	SRSF9	CLDN22
	MISP	PTPRG-AS1	HRK	DKFZP434I0714
	PLK5	PSMD6-AS2	ADAM1A	DCHS2
	C2CD4C	ADAMTS9-AS2	HCAR3	HPF1
	CIRBP-AS1	PRICKLE2-AS1	HIP1R	01-mar
	C19orf25	MAGI1-AS1	PIWIL1	NEIL3
	ATP8B3	SYNPR-AS1	CABP1	FBXW7
	CBARP	THOC7-AS1	NCOR2	TMEM144
	CSNK1G2-AS1	ARHGEF3-AS1	WSCD2	TMA16
	ODF3L2	CACNA2D3-AS1	SART3	UFSP2
	ADAMTSL5	PRICKLE2-AS2	KNTC1	DDX60
	TMPRSS9	PRICKLE2-AS3	MLEC	CDKN2AIP
	NDUFS7	SUCLG2-AS1	GIT2	TENM3
	C19orf35	LOC101929159	NUAK1	LRP2BP
	WASH5P	C3orf67-AS1	RBM19	PDGFC
	ONECUT3	ADAMTS9-AS1	RNF10	FSTL5
	MEX3D	MIR548BB	NR1H4	STOX2
	LINC01002	LOC105377102	SH2B3	CFAP97
	PRSS57	LOC105377105	ARPC3	FNIP2
	LINGO3	FAM3D-AS1	MPHOSPH9	SH3RF1

cytoband	19p13.3	3p14.1	12q24.23	4q32.2
genes in wide peak	FAM138A	LOC105377143	CAMKK2	RXFP1
	FAM138C	LOC105377146	ZNF268	SPCS3
	LOC100288123	ARF4-AS1	TRAFD1	TRAPPC11
	MIR1909		TMED2	CENPU
	MIR1302-2		ERP29	HAND2-AS1
	MIR1227		CKAP4	MAP9
	MIR1302-9		GCN1	WWC2
	MIR1302-10		SDS	CEP44
	MIR3187		RAB35	SNX25
	MIR1302-11		SNRNP35	MND1
	MIR4321		PRDM4	TKTL2
	MIR4745		CIT	CBR4
	RNU6-7		PWP1	FHDC1
	MIR6789		FICD	LOC90768
	MIR7108		FZD10	DDX60L
	RNU6-2		MLXIP	NAF1
			RPH3A	WDR17
			P2RX2	ZFP42
			FBXO21	SPATA4
			SETD1B	ENPP6
			ANKLE2	ASB5
			CUX2	SH3D19
			KIAA1033	WWC2-AS2
			MED13L	FAM218A
			SIRT4	C4orf45
			ABCB9	DCLK2
			ISCU	TRIM60
			RIMBP2	RBM46
			ATP6V0A2	C4orf46
			CORO1C	TIGD4
			FBXW8	TMEM154
			HSPB8	TMEM192

cytoband	19p13.3	3p14.1	12q24.23	4q32.2
genes in wide peak			RNU4-2	RWDD4
			RNU4-1	PRIMPOL
			HCAR1	TRIML2
			UTP20	CCDC110
			IFT81	CYP4V2
			FAM216A	F11-AS1
			HCFC2	LINC01098
			SYCP3	RNF175
			CHST11	LOC339975
			GALNT9	TRIML1
			CCDC53	LOC340017
			GPN3	PRSS48
			GLTP	ANKRD37
			MAPKAPK5-AS1	LOC389247
			ARL6IP4	TRIM61
			TAOK3	HELT
			POP5	LINC01060
			ANAPC5	FAM92A1P2
			ANAPC7	LOC441052
			TRIAP1	C4orf47
			NT5DC3	GALNTL6
			VPS29	FRG2
			TPCN1	SLED1
			SSH1	FLJ38576
			RHOF	MIR578
			VSIG10	LINC00290
			TESC	LOC728175
			PARPBP	FAM160A1
			RIC8B	LOC729870
			APPL2	MIR3945HG
			SBNO1	CLDN24
			DRAM1	DUX4

cytoband	19p13.3	3p14.1	12q24.23	4q32.2
genes in wide peak			SVOP	MIR1305
			STAB2	LOC100419170
			ZCCHC8	DBET
			POLR3B	MIR548T
			CHFR	MIR3140
			WSB2	MIR4276
			DIABLO	MIR3945
			CHPT1	MIR3688-1
			PITPNM2	LINC01207
			EP400	APELA
			DHX37	LOC100506085
			FBRSL1	LOC100506107
			DDX55	LOC100506122
			TRPV4	LINC01093
			LHX5	LOC100506272
			SUDS3	MIR4455
			C12orf43	MIR4453
			VPS33A	MIR4454
			RSRC2	MIR3688-2
			AACS	LOC100996286
			NUP37	LOC101928052
			DDX54	LOC101928131
			NOC4L	LINC01179
			GNPTAB	LINC01612
			B3GNT4	LOC101928314
			TCTN1	LOC101928509
			OGFOD2	LOC101928551
			VPS37B	LOC101928590
			C12orf49	LINC01099
			TCTN2	WWC2-AS1
			NAA25	LINC01262
			SLC8B1	LOC101930370

cytoband	19p13.3	3p14.1	12q24.23	4q32.2
genes in wide peak			RNF34	DEAR
			CCDC92	MIR6082
			MTERF2	LOC102723766
			PUS1	LOC102724776
			ACAD10	LVCAT8
			GLT8D2	LOC105377590
			KCTD10	LINC01596
			SLC41A2	
			TCHP	
			COQ5	
			HVCN1	
			SRRM4	
			CCDC62	
			KDM2B	
			UNC119B	
			USP30	
			ORAI1	
			RNFT2	
			FAM222A	
			RITA1	
			FAM222A-AS1	
			ANKRD13A	
			TMEM116	
			UBE3B	
			TMEM263	
			C12orf65	
			TMEM132C	
			BICDL1	
			SDSL	
			TMEM132B	
			IQCD	
			LINC01257	

cytoband	19p13.3	3p14.1	12q24.23	4q32.2
genes in wide peak			C12orf45	
			TMEM132D	
			SLC15A4	
			ASCL4	
			BTBD11	
			SPIC	
			ANO4	
			ALKBH2	
			FOXN4	
			SPPL3	
			BRI3BP	
			RFLNA	
			ZNF664	
			TMEM120B	
			WDR66	
			GLT1D1	
			RAD9B	
			FAM109A	
			LINC00934	
			ALDH1L2	
			SLC5A8	
			PPTC7	
			CCDC63	
			CCDC60	
			PGAM5	
			RILPL2	
			DNAH10	
			PLBD2	
			TTC41P	
			LRRC43	
			TCP11L2	
			TBX5-AS1	

cytoband	19p13.3	3p14.1	12q24.23	4q32.2
genes in wide peak			LOC283352	
			ADGRD1	
			MORN3	
			GAS2L3	
			LINC00485	
			MYO1H	
			HECTD4	
			KSR2	
			GATC	
			HNF1A-AS1	
			DDX51	
			MMAB	
			HCAR2	
			TMEM119	
			LOC338797	
			LINC01089	
			EP400NL	
			RILPL1	
			C12orf42	
			IL31	
			C12orf75	
			CFAP73	
			TMEM233	
			KMT5A	
			LINC00944	
			C12orf76	
			LINC00939	
			FLJ37505	
			LOC414300	
			LOC440117	
			FZD10-AS1	
			EID3	

cytoband	19p13.3	3p14.1	12q24.23	4q32.2
genes in wide peak			MAP1LC3B2	
			ANHX	
			SNORA49	
			MIR619	
			MIR620	
			C12orf73	
			LOC728739	
			LOC100128554	
			LOC100130238	
			LINC01405	
			USP30-AS1	
			LOC100190940	
			LINC00173	
			LOC100287944	
			ZNF605	
			MIR1302-1	
			MIR1178	
			MIR4304	
			MIR3612	
			MIR3652	
			MIR3922	
			MIR3657	
			MIR3908	
			LOC100505978	
			LINC01234	
			LOC100506551	
			PXN-AS1	
			NRAV	
			LOC100506691	
			LRCOL1	
			CLIP1-AS1	
			LOC100507091	

cytoband	19p13.3	3p14.1	12q24.23	4q32.2
genes in wide peak			LINC00943	
			ZNF664-FAM101A	
			MIR4498	
			MIR4496	
			MIR4419B	
			MIR4472-2	
			MIR4700	
			MIR4497	
			MIR5188	
			MIR548AQ	
			LINC00507	
			LOC100996671	
			LOC100996679	
			ZNF891	
			LOC101593348	
			LOC101927415	
			LOC101927464	
			LOC101927592	
			LOC101927616	
			LOC101927637	
			LOC101927694	
			LOC101927735	
			LINC01486	
			TESC-AS1	
			LOC101928416	
			LOC101928530	
			LOC101928597	
			LOC101929058	
			LOC101929084	
			CASC18	
			LOC101929162	
			MIR6760	

cytoband	19p13.3	3p14.1	12q24.23	4q32.2
genes in wide peak			MIR6761	
			MIR6762	
			MIR6861	
			MIR7107	
			MIR7641-2	
			MIR6763	
			MIR6880	
			MIR7106	
			MIR8072	
			THRIL	
			LINC01498	
			LHX5-AS1	
			LINC00508	
			LOC105369945	
			KCCAT198	
			LOC105369980	
			LOC105370014	
			LOC105370016	
			LOC105370024	
			LOC105370068	
			CRAT8	
			XLOC_009911	
			LACAT8	
			LOC107161159	

Genes included in significant GISTIC deletion peaks

cytoband	8p12	10q24.32	15q21.1	2q36.3
q value	0,18973	0,18973	0,18973	0,23137
residual q value	0,18973	0,18973	0,18973	0,23137
wide peak boundaries	chr8:25507445-37407456	chr10:80645499-13379742	chr15:1-57608540	chr2:207265195-242193529
genes in wide peak	ADRA1A	hsa-mir-3158-1	ACTC1	hsa-mir-1244-1
	BNIP3L	ACADSB	APBA2	AAMP

cytoband	8p12	10q24.32	15q21.1	2q36.3
genes in wide peak	CHRNA2	ACTA2	B2M	ACADL
	CLU	ADAM8	NBEAP1	AGXT
	DPYSL2	ADD3	BUB1B	ALPI
	DUSP4	ADRA2A	CAPN3	ALPP
	EPHX2	ADRB1	CHRM5	ALPPL2
	EXTL3	FAS	CHRNA7	ATIC
	PTK2B	ARL3	CKMT1B	KIF1A
	GSR	BMPR1A	CYP19A1	BARD1
	GTF2E2	BNIP3	DUT	BCS1L
	NRG1	CASP7	EPB42	BOK
	PNOC	ENTPD1	FBN1	CHRND
	PPP2CB	CHUK	FGF7	CHRNG
	PPP2R2A	ABCC2	GABPB1	COL4A3
	WRN	COL17A1	GABRA5	COL4A4
	FZD3	COX15	GABRB3	COL6A3
	UBXN8	CPN1	GABRG3	CPS1
	DCTN6	CTBP2	GALK2	CREB1
	PNMA2	CYP2C19	GANC	CRYBA2
	RBPMS	CYP2C8	GATM	CRYGA
	TRIM35	CYP2C9	GCHFR	CRYGB
	KIF13B	CYP2C18	PDIA3	CRYGC
	LEPROTL1	CYP2E1	HDC	CRYGD
	PURG	CYP17A1	ONECUT1	CYP27A1
	SCARA3	CYP26A1	IPW	DES
	SARAF	DMBT1	ITPKA	DTYMK
	ELP3	DNTT	IVD	EPHA4
	CCDC25	DOCK1	LTK	ERBB4
	INTS9	DUSP5	MAP1A	ACSL3
	PBK	ECHS1	MEIS2	FN1
	ZNF395	EMX2	MFAP1	GBX2
	TEX15	FGF8	TRPM1	GPC1
	EBF2	FGFR2	MYO5A	GPR35

cytoband	8p12	10q24.32	15q21.1	2q36.3
genes in wide peak	DUSP26	GFRA1	NDN	HDLBP
	HMBOX1	GLUD1	NEDD4	AGFG1
	RNF122	GOT1	OCA2	DNAJB2
	TTI2	PRLHR	PLCB2	HTR2B
	STMN4	GPR26	MAPK6	IDH1
	MAK16	GRK5	RAB27A	SP110
	FUT10	GRID1	RAD51	IGFBP2
	UNC5D	HABP2	RYR3	IGFBP5
	ESCO2	HELLS	SCG5	IHH
	FBXO16	HHEX	SLC12A1	CXCR1
	KCNU1	HMX2	SNRPN	CXCR2
	SCARA5	TLX1	SORD	CXCR2P1
	FAM183CP	HPS1	SPINT1	INHA
	NUGGC	HTR7	SRP14	INPP5D
	LINC00589	IDE	TCF12	IRS1
	MBOAT4	IFIT2	THBS1	KCNJ13
	SNORD13	IFIT1	TJP1	MAP2
	RBPMS-AS1	IFIT3	TP53BP1	MYL1
	MIR548H4	INPP5A	TYRO3	NCL
	MIR4287	KIF11	UBE3A	NDUFA10
	MIR3148	ABLIM1	MKRN3	02-sep
	MIR4288	LIPA	SLC30A4	NEU2
	MIR3622A	MGMT	PWAR5	NPPC
	MIR3622B	MKI67	EIF3J	PAX3
	SMIM18	MXI1	JMJD7-PLA2G4B	PDCD1
	NRG1-IT1	NDUFB8	SNAP23	PDE6D
	NRG1-IT3	NFKB2	HERC2	SERPINE2
	EXTL3-AS1	NRAP	SNURF	PPP1R7
	LOC101929450	OAT	USP8	PSMD1
	LOC101929470	PAX2	SLC28A2	PTH2R
	LOC101929550	PDE6C	CCPG1	PTMA
	MIR6842	PGAM1	COPS2	PTPRN

cytoband	8p12	10q24.32	15q21.1	2q36.3
genes in wide peak	MIR6843	PITX3	TGM5	SNORD20
	MIR7641-2	PNLIP	PIGB	RPE
	LINC01288	PNLIPRP1	PPIP5K1	RPL37A
		PNLIPRP2	AQR	SAG
		PPP1R3C	SECISBP2L	CCL20
		HTRA1	ARHGAP11A	SLC4A3
		PSD	LCMT2	SLC11A1
		PTEN	SLC12A6	SP100
		PTPRE	BCL2L10	SPP2
		ALDH18A1	RASGRP1	TNP1
		RBP4	SERF2	TNS1
		RGR	GNB5	TUBA4A
		RGS10	ARPP19	VIL1
		RPL21	SLC27A2	WNT6
		SCD	GPR176	XRCC5
		SFRP5	CHP1	ZNF142
		FBXW4	OIP5	FZD5
		SLC18A2	BAHD1	SCG2
		SLIT1	FAN1	CUL3
		SNCG	CEP152	DGKD
		TAF5	MAPKBP1	STK16
		TCF7L2	GOLGA8A	PER2
		TECTB	RTF1	CDK5R2
		TIAL1	CYFIP1	CNOT9
		TLL2	MGA	LRRFIP1
		UROS	DMXL2	GPR55
		WNT8B	VPS39	TRIP12
		XPNPEP1	FAM189A1	ECEL1
		SHOC2	AP4E1	EIF4E2
		ADAM12	CCNDBP1	TTLL4
		UTF1	EID1	HDAC4
		LIPF	NPAP1	FARP2

cytoband	8p12	10q24.32	15q21.1	2q36.3
genes in wide peak		EIF3A	HYPK	FARSB
		GBF1	TMEM87A	ABCB6
		LDB1	RPAP1	ARPC2
		BTRC	DKFZP434L187	ARL4C
		CH25H	PYGO1	RAMP1
		PKD2L1	BLOC1S6	SPEG
		BTAF1	GREM1	LANCL1
		PAPSS2	RPUSD2	NMUR1
		INA	TUBGCP4	STK25
		PDLIM1	SCG3	COPS8
		SMC3	TMOD3	CAPN10
		NEURL1	TMOD2	SP140
		BUB3	EHD4	IKZF2
		LGI1	DUOX2	PASK
		NOLC1	MYEF2	ATG4B
		GSTO1	NDUFAF1	OBSL1
		BAG3	RSL24D1	DNPEP
		MINPP1	NUSAP1	SH3BP4
		KIF20B	EMC4	KCNE4
		SH3PXD2A	SPTBN5	NGEF
		FAM53B	CTDSPL2	SNORD82
		SLK	KLF13	PNKD
		ZNF518A	DUOX1	SNED1
		DCLRE1A	MAGEL2	GIGYF2
		FRAT1	DLL4	TRAF3IP1
		ACTR1A	INO80	ABCA12
		SMNDC1	ZNF280D	CNPPD1
		NPM3	TRPM7	STK36
		GLRX3	PPP1R14D	CPS1-IT1
		RPP30	MTMR10	GMPPA
		DPYSL4	ZNF770	SMARCAL1
		TACC2	FLJ10038	ANO7

cytoband	8p12	10q24.32	15q21.1	2q36.3
genes in wide peak		SORBS1	HAUS2	PRLH
		ERLIN1	RMDN3	THAP4
		LBX1	DNAJC17	ANKMY1
		NRG3	MNS1	SCLY
		MGEA5	NOP10	ASB1
		TUBGCP2	MYO5C	CAB39
		PRDX3	NSMCE3	PRKAG3
		ADIRF	FAM214A	UGT1A10
		VAX1	EMC7	UGT1A8
		ATE1	PAK6	UGT1A7
		LDB3	DTWD1	UGT1A6
		SEC23IP	KNL1	UGT1A5
		RAB11FIP2	AVEN	UGT1A9
		CPEB3	ATP10A	UGT1A4
		INPP5F	GJD2	UGT1A1
		NT5C2	STARD9	UGT1A3
		PDCD11	VPS18	FEV
		SORCS3	SQRDL	PID1
		WAPL	ZNF106	ATG16L1
		PPRC1	RFX7	ANKZF1
		FAM175B	SPATA5L1	USP40
		RRP12	CHAC1	HJURP
		TBC1D12	KATNBL1	HES6
		DNMBP	ATP8B4	ASIC4
		FRAT2	WDR76	DOCK10
		IFIT5	TMEM62	MREG
		DPCD	SEMA6D	PECR
		SEC31B	SPG11	C2orf83
		ATRNL1	ELL3	MFF
		EDRF1	NIPA2	ACKR3
		LRIT1	C15orf48	RNPEPL1
		TCTN3	C15orf41	04-mar

cytoband	8p12	10q24.32	15q21.1	2q36.3
genes in wide peak		C10orf12	SPPL2A	WDFY1
		ANKRD2	ZFYVE19	NYAP2
		CNNM1	CGNL1	USP37
		MYOF	FRMD5	CTDSP1
		ANKRD1	DISP2	GAL3ST2
		GHITM	CHRFAM7A	TMBIM1
		PDCD4	ULK4P3	RAB17
		VENTX	ULK4P1	RNF25
		R3HCC1L	ARHGAP11B	COPS7B
		POLL	DPH6	MRPL44
		BLNK	C15orf57	TRPM8
		KCNIP2	KNSTRN	ATG9A
		CUZD1	BMF	MLPH
		CALY	SHF	FAM134A
		EXOSC1	DUOXA1	GLB1L
		CALHM2	SNORD107	SPAG16
		CUTC	CHST14	CHPF
		PLCE1	CASC4	IQCA1
		CHST15	TUBGCP5	NHEJ1
		SUFU	TGM7	FAM124B
		ACSL5	CATSPER2	TM4SF20
		PANK1	LEO1	C2orf54
		CCSER2	NIPA1	TUBA4B
		EXOC6	PLA2G4E	ARMC9
		FAM35A	TRIM69	EFHD1
		CCNJ	PWAR1	SPHKAP
		05-mar	TERB2	WNT10A
		ZRANB1	LOC145783	SLC19A3
		CFAP46	C15orf65	ILKAP
		NSMCE4A	LOC145845	ITM2C
		CNNM2	TMCO5A	RHBDD1
		WBP1L	ZSCAN29	ING5

cytoband	8p12	10q24.32	15q21.1	2q36.3
genes in wide peak		CCDC186	TTBK2	PLCD4
		CRTAC1	CDAN1	MGC16025
		CEP55	STRC	TMEM169
		CWF19L1	DYX1C1	DNER
		RNLS	CSNK1A1P1	B3GNT7
		PI4K2A	OTUD7A	SP140L
		HIF1AN	SPRED1	LOC93463
		WDR11	PGBD4	STK11IP
		SLF2	ADAL	MOGAT1
		DHX32	EXD1	AGAP1
		PPP2R2D	FSIP1	TWIST2
		TDRD1	RHOV	DIS3L2
		BCCIP	FAM227B	NEU4
		C10orf2	UBR1	AP1S3
		TM9SF3	PATL2	SGPP2
		ENTPD7	LPCAT4	SPATA3
		AS3MT	PLA2G4F	TMEM198
		STAMBPL1	LRRC57	ZFAND2B
		GPAM	LYSMD2	FBXO36
		SHTN1	NUTM1	MTERF4
		FAM160B1	WDR72	UBE2F
		SEMA4G	HMGN2P46	OTOS
		PLEKHA1	SLC24A5	COPS9
		AVPI1	PRTG	OR6B3
		HPSE2	LINC00926	LOC150935
		FAM204A	LOC283683	PKI55
		LHPP	GOLGA6L2	KANSL1L
		MMS19	OR4N4	LINC01107
		NOC3L	LOC283710	LOC151174
		IKZF5	FAM98B	ERFE
		ZDHHC6	PLA2G4D	METTL21A
		CUEDC2	HERC2P3	CCNYL1

cytoband	8p12	10q24.32	15q21.1	2q36.3
genes in wide peak		FBXL15	GOLGA6L1	CCDC140
		C10orf76	GOLGA8G	SLC23A3
		HPS6	GOLGA8IP	LINC00608
		MMRN2	SNORD108	GPBAR1
		MFSD13A	SNORD109A	SLC16A14
		MCMBP	SNORD115-1	LOC151475
		C10orf95	WHAMMP3	LINC00471
		PLEKHS1	GLDN	LOC151484
		PDZD7	FMN1	MSL3P1
		OBFC1	SNORD64	DAW1
		C10orf88	PWAR4	C2orf57
		UBTD1	PWARSN	PIKFYVE
		CFAP43	LINC01193	TIGD1
		LRRC27	MRPL42P5	LOC200772
		TNKS2	USP50	CFAP65
		TRIM8	TEX9	C2orf72
		KAZALD1	IGHV1OR15-1	RTP5
		SFXN3	C15orf52	LOC285095
		SLC25A28	TNFAIP8L3	UNC80
		ELOVL3	GOLGA8EP	RUFY4
		MARVELD1	OR4M2	DUSP28
		PCGF6	OR4N3P	MROH2A
		LOXL4	HERC2P10	ESPNL
		ZDHHC16	SHC4	ECEL1P2
		PCGF5	CTXN2	SPATA3-AS1
		ADGRA1	HERC2P2	CATIP
		LZTS2	C15orf53	RBM44
		LCOR	C15orf54	AQP12A
		NKX6-2	DUOXA2	KLHL30
		MRPL43	MIR211	PLEKHM3
		AFAP1L2	NF1P2	C2orf80
		PYROXD2	GOLGA6L22	RESP18

cytoband	8p12	10q24.32	15q21.1	2q36.3
genes in wide peak		USMG5	HERC2P9	C2orf82
		ATAD1	WHAMMP2	OR6B2
		ARHGAP19	GOLGA8B	ASB18
		KNDC1	EIF2AK4	FLJ43879
		ITPRIP	CATSPER2P1	VWC2L
		OLMALINC	UNC13C	MIR149
		MTG1	LINC00929	MIR153-1
		CDHR1	CKMT1A	MIR26B
		BBIP1	SERINC4	DNAJB3
		FANK1	C15orf62	LOC440934
		OPALIN	GOLGA8N	MIR375
		SYCE1	C15orf56	LOC643387
		OPN4	PHGR1	LINC00607
		HOGA1	EIF3J-AS1	LOC646736
		SORCS1	CHEK2P2	PRSS56
		PRAP1	LOC646214	AQP12B
		ZNF511	CXADRP2	SNORA75
		C10orf90	PDCD6IPP2	LOC654841
		BTBD16	IGHV1OR15-3	SCARNA6
		FAM24A	REREP3	SCARNA5
		PSTK	GOLGA8S	MIR562
		PIK3AP1	GOLGA8J	D2HGDH
		MORN4	GOLGA8T	LOC728323
		ZFYVE27	GOLGA8K	DIRC3
		MMP21	GOLGA8M	LOC100129175
		FRA10AC1	MIR626	PP14571
		ANKRD22	MIR627	LOC100130451
		SFXN2	MIR628	LOC100286922
		PDZD8	ANP32AP1	MIR1471
		BORCS7	GOLGA6L6	MIR1302-4
		NUDT9P1	LOC727924	MIR1244-1
		AGAP11	GOLGA8O	MIR548F2

cytoband	8p12	10q24.32	15q21.1	2q36.3
genes in wide peak		GSTO2	GOLGA6L7P	BOK-AS1
		SFR1	GOLGA8H	MIR1244-3
		CALHM3	PIN4P1	MIR1244-2
		CTAGE7P	OIP5-AS1	MIR3133
		CLRN3	GOLGA8CP	MIR3131
		PNLIPRP3	PWRN1	MIR4268
		SFXN4	PWRN2	MIR3132
		CPXM2	SNORD116-1	MIR4269
		ACSM6	SNORD116-2	AGAP1-IT1
		LIPJ	SNORD116-3	LOC100507443
		CFL1P1	SNORD116-4	IDH1-AS1
		TRUB1	SNORD116-5	UBE2F-SCLY
		VTI1A	SNORD116-8	MIR4439
		HECTD2	SNORD116-10	MIR4776-1
		FGFBP3	SNORD116-11	MIR4777
		C10orf82	SNORD116-12	MIR2467
		CACUL1	SNORD116-13	MIR4775
		NKX2-3	SNORD116-14	MIR4438
		SLC35G1	SNORD116-15	MIR4440
		CFAP58	SNORD116-16	MIR4786
		C10orf91	SNORD116-17	MIR4441
		PWWP2B	SNORD116-18	MIR5001
		LINC00858	SNORD116-20	MIR5702
		EMX2OS	SNORD116-21	MIR5703
		PLPP4	SNORD116-22	VWC2L-IT1
		PAOX	SNORD116-23	LOC100996693
		FAM24B	SNORD116-24	CAPN10-AS1
		EBF3	SNORD116-25	LINC01237
		CALHM1	SNORD115-2	LOC101927960
		CASC2	SNORD116-26	LOC101928020
		TCERG1L	SNORD116-27	LOC101928103
		HSPA12A	SNORD115-3	LOC101928111

cytoband	8p12	10q24.32	15q21.1	2q36.3
genes in wide peak		PIPSL	SNORD115-4	LINC01280
		FUOM	SNORD115-5	LOC101928327
		JAKMIP3	SNORD115-6	CATIP-AS1
		STK32C	SNORD115-7	LINC01494
		BLOC1S2	SNORD115-8	LOC101928881
		RBM20	SNORD115-10	MIR6513
		PDCD4-AS1	SNORD115-11	MIR6809
		FLJ37201	SNORD115-12	MIR6811
		LOC283038	SNORD115-13	MIR6810
		WDR11-AS1	SNORD115-14	LOC102723927
		FFAR4	SNORD115-15	LOC102724714
		KCNK18	SNORD115-16	LANCL1-AS1
		LIPM	SNORD115-17	LOC102724849
		CYP26C1	SNORD115-18	LOC102725079
		VWA2	SNORD115-20	MIR9500
		NANOS1	SNORD115-21	CATIP-AS2
		LRIT2	SNORD115-22	LINC01614
		HMX3	SNORD115-23	LOC105373876
		NHLRC2	SNORD115-25	DIRC3-AS1
		CCDC172	SNORD115-26	LOC105373878
		DMBT1P1	SNORD115-29	LINC01173
		C10orf99	SNORD115-30	
		SLC16A12	SNORD115-31	
		CC2D2B	SNORD115-32	
		ENO4	SNORD115-33	
		ARMS2	SNORD115-34	
		TEX36	SNORD115-35	
		LINC00959	SNORD115-36	
		GUCY2GP	SNORD115-37	
		NKX1-2	SNORD115-38	
		LBX1-AS1	SNORD115-39	
		C10orf120	SNORD115-41	

cytoband	8p12	10q24.32	15q21.1	2q36.3
genes in wide peak		LOC399815	SNORD115-44	
		METTL10	SNORD116-28	
		FLJ37035	SNORD116-29	
		FOXI2	SNORD115-48	
		LINC01164	SNORD115-24	
		LINC01168	SNORD115-27	
		SPRNP1	SNORD115-28	
		GOLGA7B	SNORD115-45	
		LINC01561	SNORD115-47	
		FAM45A	MIR147B	
		MIR107	LOC100128714	
		C10orf62	GABPB1-AS1	
		LINC00863	LOC100129973	
		IFIT1B	LOC100130111	
		FRG2B	SRP14-AS1	
		MIR346	ANKRD63	
		SPRN	LOC100131315	
		MIR146B	HERC2P7	
		MIR202	GOLGA8F	
		NPS	DCAF13P3	
		SCART1	GOLGA8DP	
		SNORA19	JMJD7	
		HOST2	PLA2G4B	
		FAM196A	POTEB2	
		FAM25A	WHAMMP1	
		LIPK	LOC100288637	
		LIPN	LOC100289656	
		LINC00865	MIR1233-1	
		RPL13AP6	MIR1266	
		SNORA12	MIR1268A	
		MIR607	MIR1282	
		MIR608	NDUFAF4P1	

cytoband	8p12	10q24.32	15q21.1	2q36.3
genes in wide peak		MIR609	LOC100419583	
		NUTM2A	LOC100422556	
		NUTM2D	MIR3118-3	
		NUTM2A-AS1	MIR1233-2	
		LINC00864	MIR3118-4	
		ENTPD1-AS1	MIR3118-2	
		RPL13AP5	MIR4310	
		RPEL1	MIR3942	
		TLX1NB	INAFM2	
		MIR936	RAD51-AS1	
		C10orf131	DPH6-AS1	
		PLCE1-AS1	SERF2-C15ORF63	
		ADGRA1-AS1	DYX1C1-CCPG1	
		ATE1-AS1	MIR4509-1	
		ACTA2-AS1	MIR4509-2	
		KLLN	MIR4508	
		TEX36-AS1	MIR4510	
		HECTD2-AS1	MIR4716	
		DNMBP-AS1	MIR4713	
		DUX4	MIR4712	
		KCNIP2-AS1	MIR4715	
		MIR1287	TMCO5B	
		MIR1307	MIR5701-2	
		MIR2110	MIR5701-1	
		FAS-AS1	SNORD116-30	
		MIR548E	SNORD115-46	
		MIR378C	LOC100996255	
		MIR4297	POTEB	
		MIR3157	GOLGA8R	
		MIR3158-1	LOC101927079	
		MIR4295	LOC101928042	
		MIR3158-2	LOC101928134	

cytoband	8p12	10q24.32	15q21.1	2q36.3
genes in wide peak		MIR4296	LOC101928174	
		MIR3941	LOC101928227	
		MIR3663	EHD4-AS1	
		MIR3944	PLA2G4E-AS1	
		SLIT1-AS1	LOC101928414	
		LINC00866	LINC01491	
		RPARP-AS1	PIGBOS1	
		SH3PXD2A-AS1	LINC01413	
		CFAP58-AS1	PWRN3	
		SORCS3-AS1	GABRG3-AS1	
		ADD3-AS1	MIR7973-2	
		LINC00867	MIR7973-1	
		GRID1-AS1	MIR8063	
		TNKS2-AS1	POTEB3	
		MARK2P9	LOC102725022	
		BORCS7-ASMT	PWRN4	
		ARHGAP19-SLIT1	LOC105370757	
		FAM24B-CUZD1	LOC105370792	
		MIR4680	LOC105370802	
		MIR4679-1	LOC105370829	
		MIR4483	LOC105370941	
		MIR4679-2	LOC105370943	
		MIR4678	BUB1B-PAK6	
		MIR4682		
		MIR4482		
		MIR4484		
		MIR4681		
		MIR4685		
		LINC00502		
		LINC00601		
		FANK1-AS1		
		SLC16A12-AS1		

cytoband	8p12	10q24.32	15q21.1	2q36.3
genes in wide peak		LINC01375		
		LOC101926942		
		PLCE1-AS2		
		LOC101927278		
		LINC01475		
		LINC01163		
		LINC01514		
		LOC101927419		
		LOC101927472		
		TCERG1L-AS1		
		LOC101927523		
		LOC101927549		
		LINC01166		
		MIR202HG		
		LOC101927692		
		MIR3663HG		
		FAM53B-AS1		
		EDRF1-AS1		
		NRG3-AS1		
		LINC01519		
		LOC101929646		
		LOC101929662		
		LINC01520		
		MIR6507		
		MIR6715B		
		MIR6715A		
		LOC102723665		
		NEURL1-AS1		
		LOC102724589		
		LOC103344931		
		LINC01435		
		LINC01167		

cytoband	8p12	10q24.32	15q21.1	2q36.3
genes in wide peak		LOC105378397		
		XLOC_008559		
		LOC105378430		
		LOC105378470		
		AS-PTPRE		
		SNORA87		
