# Supplementary material for: Homologous Recombination Repair Mechanisms in Serous Endometrial Cancer
Source: Cancers (Basel). 2021 Jan 12;13(2):254. doi: 10.3390/cancers13020254 (PMC7827019; doi:10.3390/cancers13020254)
Supplement: Supplementary file 1 [file cancers-13-00254-s001.zip › cancers-1045961-supplementary R1 - Copy/Supplementary Table S5.pdf]

Supplementary Table S5 Genes included in significant GISTIC amplification peaks

cytoband	8q24.21	10q22.3	6p21.1	3q26.32
q value	4,12E-05	0,0074492	0,016048	0,026013
residual q value	4,12E-05	0,0074492	0,016048	0,026013
wide peak boundaries	chr8:127725454-128643982	chr10:72587024-85038622	chr6:43727939-44345684	chr3:169257608-198295559
genes in wide peak	MYC	ADK	SLC29A1	ACTL6A
	PVT1	ANXA7	HSP90AB1	AHSG
	TMEM75	ANXA11	NFKBIE	APOD
	CASC11	CAMK2G	VEGFA	BCL6
	MIR1205	GLUD1P3	CAPN11	BDH1
	MIR1206	KCNMA1	TMEM63B	AP2M1
	MIR1207	MAT1A	AARS2	CLCN2
	MIR1204	P4HA1	MRPL14	CPN2
	MIR1208	PLAU	TCTE1	CRYGS
	LINC00824	PPP3CB	SPATS1	DGKG
		RGR	C6orf223	DLG1
		RPS24	SLC35B2	DVL3
		SFTPD	TMEM151B	ECT2
		VCL	LINC01512	EHHADH
		VDAC2	MIR4647	EIF4A2
		NDST2	LOC101929705	EIF4G1
		MBL1P	LOC101929726	EPHB3
		DLG5		ETV5
		SEC24C		MECOM
		PPIF		FGF12
		MICU1		GHSR
		NRG3		GP5
		POLR3A		HRG
		ECD		HES1
		ZSWIM8		IL1RAP
		DNAJC9		KNG1
		KAT6B		LPP

cytoband	8q24.21	10q22.3	6p21.1	3q26.32
genes in wide peak		NUDT13		MELTF
		LRIT1		MUC4
		AP3M1		NDUFB5
		GHITM		OPA1
		MRPS16		CLDN11
		DUSP13		PAK2
		CCSER2		PCYT1A
		ZMIZ1		PIK3CA
		MYOZ1		PLD1
		SYNPO2L		POLR2H
		TMEM254		PPP1R2
		TSPAN14		PRKCI
		C10orf11		MASP1
		FAM213A		PSMD2
		DYDC2		RFC4
		PLA2G12B		SNORA63
		ZNF503		RPL35A
		MCU		TRA2B
		CDHR1		ST6GAL1
		CHCHD1		SKIL
		MSS51		SLC2A2
		CFAP70		SOX2
		COMTD1		SST
		SAMD8		TERC
		DYDC1		TFRC
		EIF5AL1		THPO
		USP54		SEC62
		FUT11		FXR1
		OIT3		TP63
		LINC00858		CHRD
		TMEM254-AS1		TNFSF10
		PLAC9		EIF2B5

cytoband	8q24.21	10q22.3	6p21.1	3q26.32
genes in wide peak		ZCCHC24		USP13
		ZNF503-AS1		CLDN1
		ZMIZ1-AS1		MAP3K13
		FAM149B1		ADIPOQ
		DUPD1		RUBCN
		LRIT2		ECE2
		SH2D4B		ABCC5
		C10orf99		TNK2
		C10orf55		ALG3
		LINC00595		KCNMB2
		DNAJC9-AS1		IGF2BP2
		LINC00857		CLDN16
		LOC642361		NLGN1
		HOST2		NCBP2
		BEND3P3		TNIK
		SFTPA1		MCF2L2
		MIR606		ATP11B
		AGAP5		VPS8
		BMS1P4		ACAP2
		SFTPA2		UBXN7
		NUTM2B		RNU6-1
		LOC729815		GPR160
		DLG5-AS1		FETUB
		LOC100130698		LAMP3
		ZNF503-AS2		KCNMB3
		LINC00856		ZNF639
		BMS1P21		PEX5L
		ZSWIM8-AS1		DNAJB11
		MIR4676		DCUN1D1
		NUTM2B-AS1		KLHL24
		PPP3CB-AS1		PIGX
		LOC101929165		TBCCD1

cytoband	8q24.21	10q22.3	6p21.1	3q26.32
genes in wide peak		LOC101929234		P3H2
		KCNMA1-AS3		ABCF3
		KCNMA1-AS2		LSG1
		KCNMA1-AS1		PARL
		LOC101929574		MFN1
		NRG3-AS1		YEATS2
		LOC102723439		MYNN
		LOC102723703		EIF5A2
		LOC105378367		MCCC1
		LOC105378385		HRASLS
		LOC105378397		MRPL47
				NCEH1
				SLC7A14
				SENP2
				GNB4
				RTP4
				MAGEF1
				ZMAT3
				FNDC3B
				ATP13A3
				TBL1XR1
				LRRC31
				MAP6D1
				PHC3
				PIGZ
				SPATA16
				B3GNT5
				IQCG
				ATP13A4
				FYTTD1
				ACTRT3
				MGC2889

cytoband	8q24.21	10q22.3	6p21.1	3q26.32
genes in wide peak				LRCH3
				CEP19
				LMLN
				KLHL6
				VWA5B2
				TMEM41A
				TMEM44
				CAMK2N2
				TM4SF19
				RPL39L
				DNAJC19
				FAM131A
				ZDHHC19
				LRRC15
				FAM43A
				TMEM207
				RTP1
				TTC14
				LRRC34
				MB21D2
				XXYLT1
				CCDC50
				PYDC2
				NCBP2-AS2
				RNF168
				HTR3C
				LIPH
				HTR3D
				RPL22L1
				SLC51A
				FBXO45
				MUC20

cytoband	8q24.21	10q22.3	6p21.1	3q26.32
genes in wide peak				SENP5
				LOC220729
				LOC253573
				NAALADL2
				TCTEX1D2
				SMCO1
				SDHAP1
				UTS2B
				HTR3E
				C3orf70
				TPRG1
				LOC285389
				CCDC39
				EHHADH-AS1
				LPP-AS2
				LRRIQ4
				SAMD7
				NMRAL1P1
				RTP2
				OSTN
				ATP13A5
				SOX2-OT
				WDR53
				ANKRD18DP
				NRROS
				TMEM212
				FLJ46066
				FLJ42393
				LINC00884
				LINC00885
				MIR28
				LINC00969

cytoband	8q24.21	10q22.3	6p21.1	3q26.32
genes in wide peak				SNORD2
				SNORA4
				IGF2BP2-AS1
				GMNC
				LOC647323
				SNORA81
				SNORD66
				MIR569
				MIR570
				SDHAP2
				FAM157A
				MIR922
				MIR944
				DPPA2P3
				LOC100128164
				LINC00887
				LOC100131635
				SNAR-I
				MIR1224
				MIR1248
				MIR3137
				LINC01208
				LINC00578
				LOC100505609
				LINC00888
				LOC100505920
				MFI2-AS1
				DLG1-AS1
				TMEM44-AS1
				LOC100507391
				TM4SF19-TCTEX1D2
				MIR4448

cytoband	8q24.21	10q22.3	6p21.1	3q26.32
genes in wide peak				MIR4797
				MIR4789
				LINC00501
				MIR5588
				MIR548AQ
				NAALADL2-AS3
				LPP-AS1
				OPA1-AS1
				LMLN-AS1
				ABCC5-AS1
				FGF12-AS1
				FGF12-AS3
				NLGN1-AS1
				KLHL6-AS1
				TPRG1-AS2
				UBXN7-AS1
				TPRG1-AS1
				EIF2B5-AS1
				ADIPOQ-AS1
				TM4SF19-AS1
				ATP13A5-AS1
				TMEM212-AS1
				NAALADL2-AS2
				NAALADL2-AS1
				LINC01014
				XXYLT1-AS1
				LINC01206
				XXYLT1-AS2
				LOC101928583
				LINC01209
				LOC101928739
				PEX5L-AS2

cytoband	8q24.21	10q22.3	6p21.1	3q26.32
genes in wide peak				LOC101928882
				LOC101928992
				LOC101929106
				LOC101929130
				P3H2-AS1
				ATP13A4-AS1
				LOC101929337
				LOC101929697
				LINC01063
				RNU6-7
				MIR6828
				MIR7977
				MIR6829
				KCCAT211
				LOC102724604
				LOC102724699
				LINCR-0002
				RNU6-2
				KCNMB2-AS1
				LOC105374205
				LOC105374244
				LOC105374250
				LOC105374266
				LOC105374297
				HTR3E-AS1
				OSTN-AS1

Genes included in significant GISTIC amplification peaks

cytoband	17q12	18q11.2	14q11.2	19q12
q value	0,12943	0,13216	0,13973	0,13973
residual q value	0,12943	0,13216	0,13973	0,13973
wide peak boundaries	chr17:37666372-39694523	chr18:13982664-30683883	chr14:1-24266417	chr19:29382842-30440290
genes in wide peak	CACNB1	AQP4	hsa-mir-1201	CCNE1
	ERBB2	CDH2	ANG	URI1
	LASP1	GATA6	APEX1	ZNF536
	MLLT6	LAMA3	BCL2L2	POP4
	NEUROD2	NPC1	CEBPE	PLEKHF1
	PNMT	RBBP8	DAD1	C19orf12
	MED1	ROCK1	HNRNPC	LOC284395
	PSMB3	SNRPD1	MMP14	VSTM2B
	RPL19	SS18	MYH6	
	HNF1B	TAF4B	MYH7	
	PCGF2	RIOK3	NEDD8	
	PIP4K2B	ZNF521	PNP	
	TCAP	CABYR	NRL	
	RPL23	C18orf8	OXA1L	
	STARD3	IMPACT	PCK2	
	SOCS7	MIB1	PSMB5	
	CDK12	HRH4	PSME1	
	CWC25	CTAGE1	PSME2	
	PLXDC1	GREB1L	RABGGTA	
	ARHGAP23	CHST9	RNASE1	
	SRCIN1	TMEM241	RNASE2	
	PPP1R1B	CABLES1	RNASE3	
	TBC1D3F	ESCO1	RNASE4	
	MRPL45	OSBPL1A	RNASE6	
	FBXL20	TTC39C	SALL2	
	PGAP3	PSMA8	TEP1	
	YWHAEP7	AQP4-AS1	TGM1	

cytoband	17q12	18q11.2	14q11.2	19q12
genes in wide peak	CISD3	ANKRD29	PABPN1	
	LRRC37A11P	ZNF519	AP1G2	
	STAC2	ABHD3	SLC7A7	
	C17orf98	CYP4F35P	CPNE6	
	ARL5C	KCTD1	TOX4	
	TBC1D3C	ANKRD30B	REC8	
	LOC440434	POTEC	PARP2	
	GPR179	LINC01443	DHRS2	
	FBXO47	MIR1-2	EFS	
	SNORA21	MIR133A1	IRF9	
	TBC1D3	CXADRP3	PRMT5	
	TBC1D3H	ANKRD20A5P	TM9SF1	
	LOC100131347	LOC644669	EDDM3A	
	C17orf96	PCAT18	DHRS4	
	LINC00672	LOC729950	SUPT16H	
	MIR4726	GATA6-AS1	ACIN1	
	MIR4734	MIR302F	SLC7A8	
	MIR4727	MIR320C1	NGDN	
	TBC1D3G	MIR320C2	LRP10	
	TBC1D3K	MIR3156-2	TINF2	
	TBC1D3L	LINC01543	OR10G3	
	LOC101060389	MIR4741	OR10G2	
	MIR6779	LOC101927571	OR4E2	
	TBC1D3E	LINC01444	OR4E1	
	TBC1D3I	MIR8057	CHMP4A	
	LOC105371766	MIR133A1HG	SLC39A2	
		TTC39C-AS1	EMC9	
		LOC105372028	ZNF219	
		LOC105372038	GMPR2	
			SLC22A17	
			HAUS4	
			C14orf119	

cytoband	17q12	18q11.2	14q11.2	19q12
genes in wide peak			RNF31	
			RBM23	
			DHRS4-AS1	
			OSGEP	
			ARHGEF40	
			METTL3	
			RPGRIP1	
			NDRG2	
			HOMEZ	
			CHD8	
			CCNB1IP1	
			C14orf93	
			ABHD4	
			EDDM3B	
			CDH24	
			METTL17	
			IL25	
			THTPA	
			OR4K5	
			OR11H2	
			OR4K1	
			IPO4	
			DCAF11	
			OR4K15	
			JPH4	
			RNASE7	
			RAB2B	
			AJUBA	
			ZFHX2	
			RPPH1	
			CARMIL3	
			PPP1R3E	

cytoband	17q12	18q11.2	14q11.2	19q12
genes in wide peak			TMEM55B	
			TTC5	
			CMTM5	
			RNASE11	
			TPPP2	
			RNASE8	
			MRPL52	
			PSMB11	
			OR4K14	
			OR4L1	
			OR11H6	
			KLHL33	
			MDP1	
			FITM1	
			REM2	
			LOC254028	
			LINC00641	
			TSSK4	
			DHRS4L2	
			SNORD8	
			RNASE10	
			OR6S1	
			OR4N2	
			OR4K2	
			OR4K13	
			OR4K17	
			OR4N5	
			OR11G2	
			OR11H4	
			RNASE9	
			OR5AU1	
			POTEG	

cytoband	17q12	18q11.2	14q11.2	19q12
genes in wide peak			MIR208A	
			OR11H12	
			RNASE13	
			OR4Q3	
			OR4M1	
			RNASE12	
			DUXAP10	
			POTEM	
			LOC642426	
			LINC01296	
			ECRP	
			TMEM253	
			SNORD9	
			DHRS4L1	
			SNORD126	
			MIR208B	
			PRMT5-AS1	
			RNF212B	
			LOC100508046	
			NEDD8-MDP1	
			BCL2L2-PABPN1	
			MIR4707	
			POTEH-AS1	
			BMS1P17	
			LOC101926933	
			LOC101929572	
			LOC101929718	
			MIR6717	
			MIR7703	
			LOC102724814	
			MHRT	
			BMS1P22	

Genes included in significant GISTIC amplification peaks

cytoband	11q13.1
q value	0,22918
residual q value	0,22918
wide peak boundaries	chr11:64043444-64121820
genes in wide peak	FLRT1
	MACROD1
